# Outlier-detection for reactive machine learned potential energy surfaces

**DOI:** 10.1038/s41524-024-01473-6

**Published:** 2025-02-15

**Authors:** Luis Itza Vazquez-Salazar, Silvan Käser, Markus Meuwly

**Affiliations:** Department of Chemistry, University of Basel, Basel, Switzerland

**Keywords:** Theoretical chemistry, Physical chemistry

## Abstract

Uncertainty quantification (UQ) to detect samples with large expected errors (outliers) is applied to reactive molecular potential energy surfaces (PESs). Three methods–Ensembles, deep evidential regression (DER), and Gaussian Mixture Models (GMM)—were applied to the H-transfer reaction between *syn*-Criegee and vinyl hydroxyperoxide. The results indicate that ensemble models provide the best results for detecting outliers, followed by GMM. For example, from a pool of 1000 structures with the largest uncertainty, the detection quality for outliers is ~90% and ~50%, respectively, if 25 or 1000 structures with large errors are sought. On the contrary, the limitations of the statistical assumptions of DER greatly impact its prediction capabilities. Finally, a structure-based indicator was found to be correlated with large average error, which may help to rapidly classify new structures into those that provide an advantage for refining the neural network.

## Introduction

Detecting infrequent and/or out-of-distribution events is central to data-driven research. Fields in which such events are relevant range from finance^1^ to medicine^2^, climate^3^, weather and the natural sciences^4^. While “expected” outcomes can be typically sampled from a known, computable, and controllable distribution, infrequent (or “rare”) events can not always be easily associated with a predetermined distribution. In most cases, it is, however, the rare events that profoundly affect the development of a system, such as a crash in stock markets, a tornado in weather, or a bond-breaking/forming process in chemistry. A typical chemical bond with a stabilization energy of ~20 kcal/mol (equivalent to a lifetime of 1 s^−1^) and a vibrational frequency of 20 fs^−1^ vibrates ~10^13^ times before breaking, which makes chemical reactions a “rare event”. As the energy in the system increases for bond breaking (and bond formation) to occur, the available phase space increases in concert, and sampling all necessary regions becomes a daunting task.

Computer simulations are an indispensable part of today’s research and have become increasingly important in chemistry, physics, biology, and materials science. One particularly fruitful approach for the chemical and biological sciences are molecular dynamics (MD) simulations^5–8^ that involve the numerical integration of Newton’s equations of motion. This requires the knowledge of the underlying intermolecular interactions (the “potential energy surface” (PES)) and forces derived from them for a given atomic configuration **x**^9,10^. Ideally, those properties would be determined at the highest level of accuracy by solving the time-independent Schrödinger equation (SE). Unfortunately, this is only possible for small systems on a short time scale because the methods to solve the SE scale poorly with the system size and the method’s accuracy. This limitation can be circumvented by using atomistic potentials that directly describe the relation between the atomic positions of a molecule and its potential energy through the mapping, $f:{\{{Z}_{i},{{\bf{x}}}_{i}\}}_{i = 1}^{N}\to E({\bf{x}})$, of the atomic charges (*Z*_*i*_) and the atomic positions (**x**_*i*_) to the potential energy *E*(**x**) from which the forces can be determined as its negative gradient (*F*_*i*_ = −∇*E*(**x**)).

Over the last decade, machine learning (ML) techniques such as neural networks (NNs) and kernel methods have been used to represent PESs^9,11–14^. This originates from the methods’ ability to learn relationships from data^15^. Therefore, it is possible to parametrize/learn the described mapping from a pool of reference ab initio calculations and eventually use it for following the dynamics of a system of interest. Particularly, ML has been extensively used to represent PESs based on large, diverse, and high-quality electronic structure data^16–22^. While machine-learned potential energy surfaces (ML-PESs), sometimes also called ML potentials, not to be confused with multilayer perception, reach remarkable accuracies (orders of magnitude better than “chemical accuracy”, i.e., 1 kcal/mol) in the interpolation regime of the data set they are known to extrapolate poorly on unseen data due to their purely mathematical nature lacking any underlying functional form^23,24^. Thus, ML-PESs crucially depend on the globality of the training data, which usually requires an iterative collection/extension of a data set^9,15,25^.

On the other hand, constructing globally valid ML-PESs, in particular for chemical reactions, is still a challenging task because the reaction channels might not be known a priori and because the phase space that needs to be covered increases exponentially with the energy that is required to drive a conventional chemical reaction. This is directly related to the quality, completeness and coverage of the data set used to train the ML algorithm, in particular for NN-based representations. One way to tackle these critical aspects is through the use of uncertainty quantification (UQ) with the primary goal of detecting uncovered regions. Those regions are characterized by the presence of outliers (i.e., samples with largely different behavior than the other members of the data set^26^), which usually also have large errors. Finding such outliers or outlier regions and including samples in the training helps to increase the model’s robustness and further improves its accuracy and reliability. Particularly for reactive PESs—one of the hallmark applications of ML-based PESs—quantitatively characterizing the confidence in predicted energies and/or forces for chemically interesting regions around the transition state(s) (TS) is very valuable.

For chemical applications, different UQ techniques have been used. Common are ensemble methods for which multiple independently trained statistical models are used to obtain the average and variance of an observation^27^. Depending on the number of ensemble members, their disadvantage lies in the high computational cost they incur. Alternatively, methods based on Gaussian process regression^28^ were employed, which, however, are limited by the database size for which they can be used. Alternatives based on single-network methods with the possibility to predict the variance have been proposed, including regression prior networks^29^, mean variance (MV) estimation, or deep evidential regression (DER)^30,31^. Recently, the use of several UQ techniques (Ensembles, DER, MV estimation, and gaussian mixture models, GMM) was benchmarked primarily for near-equilibrium and non-reactive PESs^32^. Conversely, the present work trains and analyzes PESs for describing chemical reactions which necessarily sample regions far from equilibrium. The breaking and formation of chemical bonds is at the heart of chemistry and PESs suitable to describe chemical reactions in a meaningful fashion pose additional challenges including, but not limited to, discontinuities caused by multiple reaction channels or multi-reference (MR) effects. Additionally, the approach pursued here considers the prediction of multiple properties (i.e., energy, forces, dipoles) and further modifications to the DER model.

The focus of the present work is on the use of UQ methodologies to detect outliers in constructing PESs for chemical reactions. To this end, the first step of the decomposition reaction of the *syn*-Criegee intermediate (CH_3_CHOO ⟶ CH_2_CHOOH (VHP) ⟶ CH_2_CHO + OH or glycolaldehyde^21^) is considered by training, validating the quality and analyzing the error measures of the PESs based on different UQ tools. Complementary to outlier detection, an analysis of in/out-of-distribution of the samples in the test set with respect to the training distribution using a metric based on intermolecular distances was performed. As ML permeates more throughout daily life and is used in life-critical situations (e.g., self-driving cars^33^, medical diagnosis^34^, etc.), it is important to quantify whether identified outliers are related to a lack of information or to a new discovery.

In the following, the results for the characterization of the PES in the previously described ways are discussed, followed by an assessment of the error distributions, outlier detection, and in/out-distribution analysis. Next, the main findings are discussed in a broader context, and conclusions are drawn. Lastly, the methods, including data set generation, UQ methodologies, outlier detection, and inside-outside distribution metrics, are described.

## Results

### Characterization and validation of the trained PESs

First, the quality of the generated PESs was assessed to ensure that they can indeed be used for meaningful atomistic simulations which is the final goal of any ML-PES. Applying UQ and considering outlier detection is only meaningful for validated PESs. The validation of a PES is an aspect that differs from ML-based models for chemical databases because PESs need to satisfy particular conditions for meaningful exploration of chemical processes. Several different and complementary metrics were used in the following. Initially, the overall performance of the trained PESs was evaluated on a hold-out test set. Second, the capabilities of the PESs to describe characteristic regions, including minima and transition states, as well as the shape of the PESs around the minimum, were tested by computing energies and harmonic frequencies. Finally, the usefulness of the generated PESs for reactive MD simulations was assessed by following the minimum energy path (MEP), minimum dynamic path (MDP), and establishing energy conservation in MD simulations. The MEP describes the lowest energy path connecting reactants and products passing through the TS. Complementary to the MEP, the MDP^35^ provides information about the least-action reaction path in phase space

**Fig. 1:**
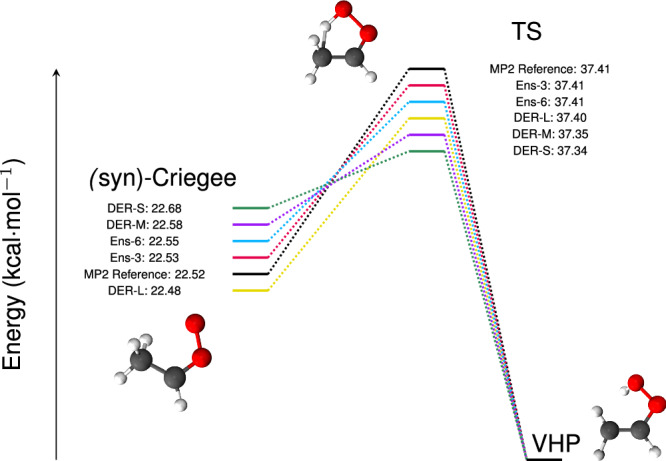
Characteristics of the stationary points of the PESs. The energy of the VHP minimum serves as a reference. The energy scale is exaggerated to better represent the differences between the methods.

The performance of all trained models was assessed on a hold-out (test) set and the MAEs and RMSEs on energies and forces are given in Table S1. While most models reach similar MAE(*E*) ≤ 1.0 kcal/mol, the performance of the forces deserves more attention and is discussed further below. An essential requirement of an ML-PES is to adequately describe geometries and relative energies of particular structures, including the minima and transition states, which are shown in Fig. 1. All models considered perform adequately to predict energies of stationary points with errors of <0.1 kcal/mol. The errors for the *syn-*Criegee structure are 0.01, 0.03, 0.16, −0.04, and 0.06 kcal/mol for Ens-3, Ens-6, DER-S, DER-L, and DER-M compared with errors lower than 0.01 kcal/mol for the TS using ensembles, and −0.07, −0.01, and 0.06 kcal/mol with DER-S, DER-L, and DER-M, respectively. The smaller error of Ens-3 compared with Ens-6 is counter-intuitive and may be a consequence of random noise in the prediction caused by, e.g., parameter initialization, convergence of the loss function, or numerical inaccuracies^36^. Complementary to the energy of the equilibrium structures, the root mean squared displacement (RMSD) between optimized geometries from the trained NN models and at the MP2 level were compared; see Fig. S4. Although the differences between the models are small, the errors of the DER-models are two orders of magnitude larger than for the ensembles, see the supporting information for further details.

Harmonic frequencies, obtained from the Hessian matrix (*H* = ∂^2^*E*/∂x^2^), characterize the shape of the PES around stationary points. The results (Fig. S5 and Tables S2–4 for *syn-*Criegee, TS and VHP) indicate that the best performers are the ensemble models and GMM with an MAE one order of magnitude lower than the DER models. Regarding the DER models, the best performer is DER-L, followed by DER-S and DER-M. DER-L displays errors between −50 cm^−1^ and 50 cm^−1^, whereby most of the frequencies below 1500 cm^−1^ were underestimated, and those above 2000 cm^−1^ (XH stretch) were overestimated. Conversely, DER-S underestimates most frequencies, showing the largest errors for the vibrations at larger frequencies. The worst-performing model, DER-M, shows a large overestimated value at ~500 cm^−1^ and a large underestimated value at high frequencies. The harmonic frequencies for the TS and for VHP follow similar trends. It is interesting to note that the large errors in the harmonic frequencies are also observed for the forces; in general, DER models have an MAE(F) one order of magnitude larger than the other three models evaluated here; see Table S1. This is a direct consequence and a limitation of the assumed normal distribution of the energies because the error model depends on energy explicitly. The forces and Hessians are derivatives of the energy expression, and the associated errors are $\propto \frac{{{\rm{Error}}}_{{\rm{Ener.}}}^{2}}{{\sigma }^{2}}$ and $\propto \frac{{{\rm{Error}}}_{{\rm{Ener.}}}^{3}-{\sigma }^{2}}{{\sigma }^{4}}$, respectively. Hence, the DER models have an inferior performance for forces and harmonic frequencies.

**Fig. 2:**
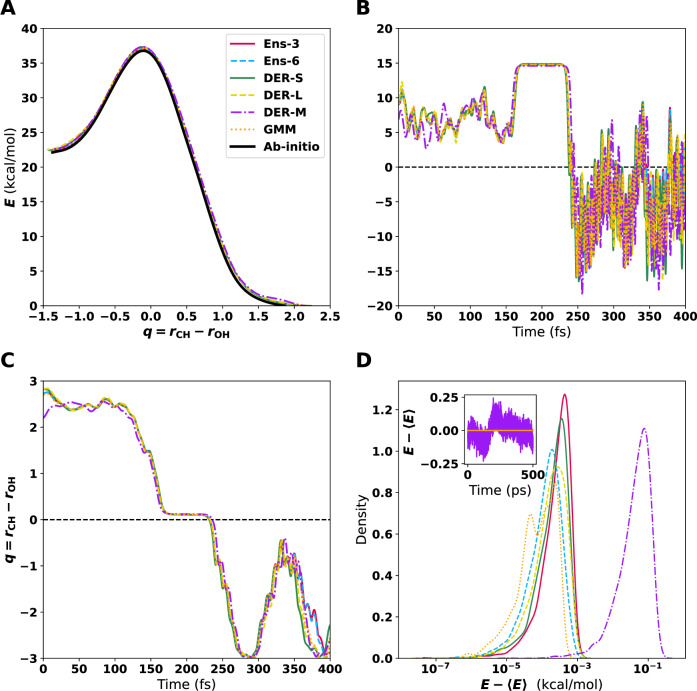
Characterization of the different PESs to describe the reaction from *s*yn-Criegee to VHP. **A** Minimum energy path (MEP) from *s*yn-Criegee to VHP. The zero energy is the corresponding value for the optimized structure of VHP. **B** Variation of the energy along minimum dynamic path (MDP) using the different ML-PESs starting from the optimized TS. **C** Time series of the reaction coordinate (*q* = *r*_CH_−*r*_OH_) from the MDP. **D** Energy distribution from MD simulations using the different PESs. Note that the *x* axis is on a logarithmic scale. Starting from *(syn)*-Criegee, the system was simulated for 500 ps with a time step of 0.1 fs. The inset shows the time series of the energy for DER-M. The high-frequency oscillations on the product side (VHP) in **B** have a period of ~10 fs corresponding to a frequency of ~3500 cm^−1^ characteristic of the OH-stretch vibration, whereas the low-frequency oscillation in **C** is due to the azimuthal rotation of the -OH group.

Figure 2A shows the MEP for the different models. All MEPs are within <0.5 kcal/mol on each of the points sampled. Therefore, despite the differences in how errors are handled and their magnitude for each model, the MEP determined from the PESs are consistent with one another. The MDPs (see Fig. 2B), initiated from the TS, were determined with an excess energy of 10^−4^ kcal/mol. The TS structure is stabilized because it is a 5-membered ring and because little excess energy was used for the MDP. VHP is observed after 225 fs accompanied by pronounced oscillations in the potential energy primarily due to the highly excited OH-stretch. Overall, the time traces for potential energy (Fig. 2B), one possible reaction coordinate *q* = *r*_CH_ − *r*_OH_ (Fig. 2C), and all atom-atom separations in Fig. S6 are rather similar for the 6 PESs. The notable exception concerns primarily DER-M (purple), for which the energy and the C1-H2 and C2-H3 separations deviate noticeably from the other 5 models; see Fig. S6.

Lastly, *NVE* simulations with all six models were carried out; see the methods section for details on these simulations. The MD simulations were run for 500 ps with a time step of Δ*t* = 0.1 fs. The results show that energy is conserved within ~0.1 kcal/mol or better for all PESs, see Fig. 2D. Importantly, no drift was found for all PESs, except for DER-M.

### Error distributions and outlier detection

Next, the errors, their distributions, and the detection of outliers are discussed. For PESs, specifically, it is desirable that a trained model accurately predicts the energies across a wide range of geometries and energies. This is one of the hallmarks of a robust ML model for intermolecular interactions and is a prerequisite for its extrapolation capabilities. The data set considered contains structures for *(syn)*-Criegee, VHP, and the corresponding TS. Residual plots were used to describe how the signed error Δ*E* = *E*_Ref_ −*E*_Pred_, is distributed for energies between −700 and −300 kcal/mol.

#### Ensembles

**Fig. 3:**
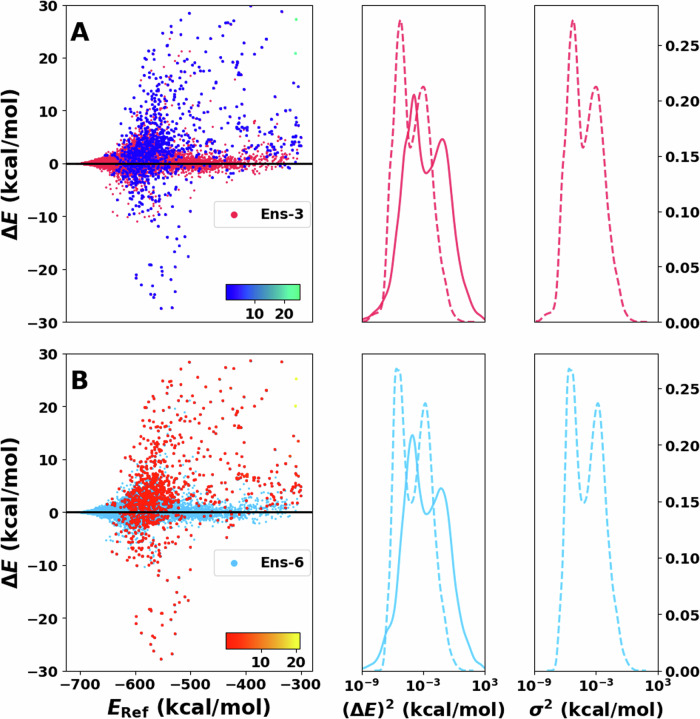
Performance of the Ens-3 and Ens-6 models on the test set. **A**, **B** on the left show residual plots of the error between reference and prediction. The 1000 energies with the largest variance are shaded with a different color and directly reflect the model’s capability to detect outliers. The corresponding color bar (blue to green/red to yellow) represents the scale of the variance. Note that the variance of most of the test samples is at the lower end of the color bar. Squared error distribution (solid lines) and variance distributions (dotted lines) are shown in the centre next to **A**, **B** for comparison. Complementary to this is the variance distribution shown on the right of both panels. Notice that the *x* axis on the centre and right are in logarithmic scale.

Figure 3 shows the performance of the ensembles. Here, the errors $\Delta {E}$ range from −30 to 30 kcal/mol, with most errors near the centre (i.e., Δ*E* = 0). The region with the lowest energy (*E* < −650 kcal/mol) has higher accuracy with no noticeable high-error structures. The next region, between −650 and −500 kcal/mol, have the largest number of high-error structures broadly spread between positive and negative errors. For higher energies (above −500 kcal/mol), a small spread of the errors with few significant outliers is found. It can be noticed that the region with more outliers is close in energy to the transition state; therefore, the structures are expected to have larger deformation than the other regions. This is related to the fact that the training data set was created to reproduce adequately the hydrogen transfer.

The distributions of the squared error (*P*((Δ*E*)^2^)) and the variance (*P*(*σ*^2^)) in Fig. 3 are both rather sharp and centered ~0. Using a logarithmic scale further clarifies the structure of these distributions. The bimodal nature of *P*((Δ*E*)^2^) and *P*(*σ*^2^) is the first distinctive feature. In addition, the predicted variance largely matches the squared error distribution (Fig. 3 centre). The distributions agree nearest to their centre. However, the height of the distribution is larger for *P*(*σ*^2^) than for *P*((Δ*E*)^2^). Furthermore, the tails of *P*(*σ*^2^) decay faster than for *P*((Δ*E*)^2^). This is reflected in fewer samples labeled with large variance than the number of structures with large squared error. It should be noted that for outlier detection the overall shape of *P*((Δ*E*)^2^) and *P*(*σ*^2^) (single Gaussian vs. bi- or multimodal) is inconsequential because invariably, the instances with the largest Δ*E*, which are in the high-Δ*E*-tail, are of primary interest.

#### Deep evidential regression

**Fig. 4:**
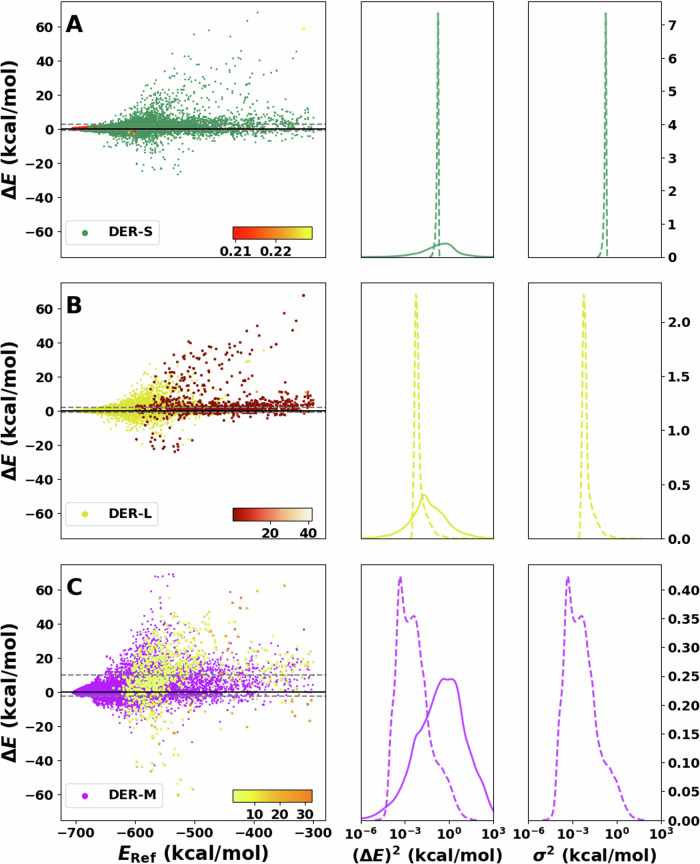
Performance of the different versions of PhysNet-DER on the test set. **A**–**C** On the left show residual plots of the error between reference and inference for DER-S, DER-L, and DER-M, respectively. The 1000 points with the largest variance are shaded with a different color (red, orange, and yellow from top to bottom) and directly reflect the model’s capability to detect outliers. The corresponding color bar represents the scale of the values. Squared error distribution (solid lines) and variance distributions (dotted lines) are shown in the center next to **A**–**C** for comparison. Complementary to this is the variance distribution shown on the right of both panels. Notice that the *x* axis on the centre and right are in logarithmic scale.

The results for the predictions of the DER models are displayed in Fig. 4. For DER-S, the errors are spread between −60 and 60 kcal/mol, and the variances vary between 2 × 10^−3^ to 9 × 10^−3^ kcal/mol with a single sharp peak around 10^−2^ kcal/mol, i.e., the same uncertainty for nearly all predictions. This aligns with the previously discussed problems of DER^37^ reported models which improve the quality of the predictions by increasing their uncertainty. The small variances across the test set indicate that adding forces and dipole moments to the loss functions renders the model overconfident. One possible explanation is that terms depending on forces, charges, and dipoles in Equation (2) to DER-S act as extra regularizers to the evidence of incorrect predictions, akin to the ${{\mathcal{L}}}^{R}(x)$ term, during training of the NN. Hence, the variance predicted by DER-S loses its capability to detect outliers. Furthermore, DER-S tends to underestimate the energies with a larger population on the positive side of the Δ*E*. Finally, the squared error, centered around 10^0^, is spread over a wide range from 10^−4^ to a few tens of kcal/mol.

Next, DER-L is considered (see Fig. 4B) for which the error increases with the energy. Complementary, the variance is high for structures with positive Δ*E* (red points). The variance distribution is sharply peaked and centered ~10^−3^, showing some overlap with *P*((Δ*E*)^2^), whereas *P*((Δ*E*)^2^) is unimodal and centered at 10^−1^ kcal/mol. However, the tails are wide and extend to 10^2^ kcal/mol. As for DER-S, the center of mass of *P*(*σ*^2^) is between 1 or 2 orders of magnitude smaller than *P*((Δ*E*)^2^), indicating that DER-L is overconfident about its predictions. It is also noted that DER-L is biased to identify predictions that underestimate the energy (i.e., positive Δ*E*) as high-error structures.

Finally, DER-M (Fig. 4C) features a large dispersion of the predicted error around the energy range considered in this work. Predictions deteriorate quickly for low-energy configurations with almost no points near the diagonal. *P*((Δ*E*)^2^) is centered ~1 kcal/mol and extends from 10^−2^ to 10^2^ kcal/mol with some overlap with the bimodal *P*(*σ*^2^) centered at ~10^−4^, around four orders of magnitude smaller than *P*((Δ*E*)^2^). Regarding the detection of outliers, it is found that samples that underestimate the energy display a large variance. On the technical side, it has been found that optimization of multidimensional Gaussian models, such as DER-M, can be numerically challenging because the NN-prediction of the covariance matrices can be numerically unstable^38–40^.

Differences between the three flavors of DER were noticeable. Firstly, DER-M performs worst on energy predictions with a poor quality of the underlying PES. On the other hand, DER-S and DER-L show a similar distribution of errors; see Fig. 4. *P*(*σ*^2^) for DER-M is bimodal and considerably broader than for the other two models, which show a single sharp peak. The width of *P*(*σ*^2^) for DER-M increases the overlap with the (Δ*E*)^2^ distribution and, therefore, is more likely to identify outliers than the other two DER models. Unfortunately, the variance values predicted by DER-M underestimate the error by 2 to 3 orders of magnitude. From these results, DER-L is the best performer with the smallest MAE among the DER models and medium quality for the variance estimation.

#### Gaussian mixtures models

**Fig. 5:**
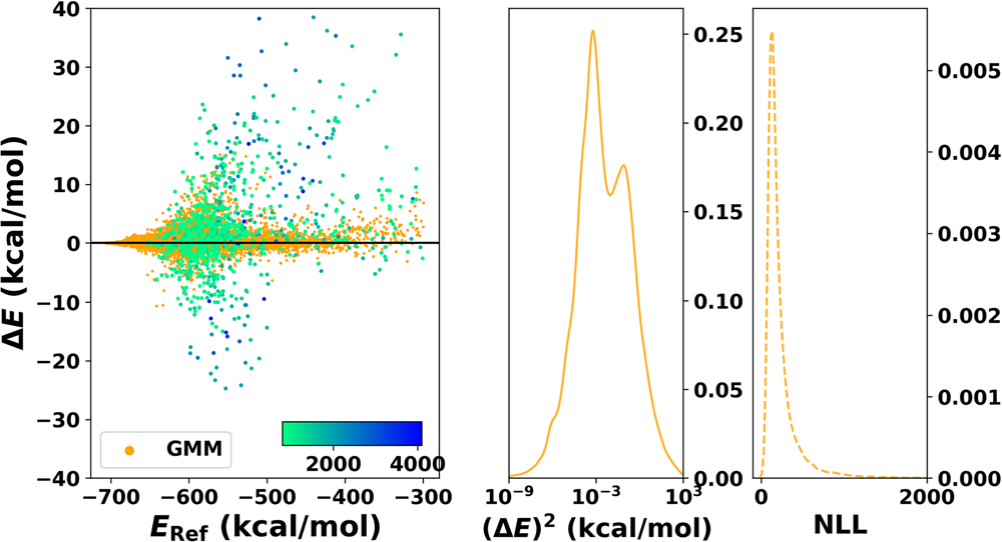
Performance of the PhysNet-GMM on the test set. Residual plot of the error between reference and production is shown on the left. The 1000 points with the largest negative log-likelihood (NLL) value are shaded with a different color and directly reflect the model’s capability to detect outliers. The corresponding color bar represents the scale of the values. The panel in the centre shows the squared error distribution. Note that the x-axis of the centre panel is in logarithmic scale for clarity. The panel on the right displays the distribution of the NLL, which is used to quantify the uncertainty. Note that the axis of the NLL is cut for clarity.

Finally, for the GMM (Fig. 5), the dispersion of the error increases as the energy increases. Specifically, the largest errors occur for the highest energies. For the errors, it is found that they are more evenly distributed in the over- (Δ*E* < 0) and under-predicted (Δ*E* > 0) regions. On the other hand, *P*((Δ*E*)^2^) features a bimodal distribution centered at 10^−3^ with extended tails up to 10^3^ with the NLL peaked at low values of NLL and decays rapidly for increasing NLL.

The error analysis carried out so far indicates that outlier detection is challenging in general. While the high-error structures are reliably captured, particularly for Ens-3, Ens-6, Der-L, and GMM, they also falsely classify structures with low errors as outliers. In this work, outlier detection capabilities of the models are evaluated using the accuracy metric defined in Equation (12) and the classification procedure described in the methods section.

**Fig. 6:**
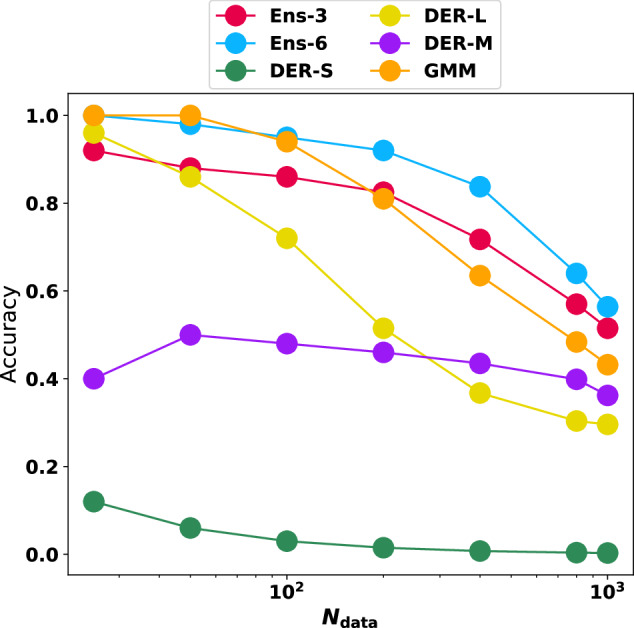
Reliability of outlier detection for the different strategies. Given the 1000 structures with the largest variance/uncertainty, it is evaluated whether they correspond to the structures that also have the largest errors from comparison with reference data for *N*_data_ = [25, 50, 100, 200, 400, 800, 1000]. i.e., it is evaluated whether the *N*_data_ structures with the actual highest errors are contained in the 1000 that are predicted to have high errors.

First, the number of structures with large variance was determined, and the magnitude of the error was assessed. Instead of defining a cutoff value—which is necessarily arbitrary to some extent—the present work considers and analyzes predictions with the largest uncertainty value. This makes the analysis independent of particular numerical choices. The minimum and maximum values for squared errors and variance/NLL are summarized in Table S5. Figure 6A shows the results for the 1000 structures with the largest predicted variance. The results indicate that as the number of structures with large errors sought increases, the probability of finding them among the top 1000 with large variance decreases. Overall, the best-performing model is Ens-6, closely followed by Ens-3 and GMM. The three DER models behave quite differently from one another. First, DER-S has a poor performance and approaches zero ability to detect outliers. Next, DER-L is very good at detecting extreme outliers, performing even better than Ens-3 for *N*_data_ = 25. However, its performance decays quickly and is the second worst after DER-S for *N*_data_ = 1000. Finally, DER-M has an almost linear performance, meaning its capability predictions are constant, independent of the number of samples.

One interesting aspect of Fig. 6A is that for the extreme cases (i.e. detecting the 25 samples with the largest error), four models (Ens-3, Ens-6, DER-L, and GMM) have a probability higher than 80% for detecting those extreme values. This trend continues for the ensemble models and GMM up to *N*_data_ = 200 beyond which the accuracy decays for all models. This can be understood because the task at hand is harder to solve as the number of required samples to identify increases. This is clearly observed in Fig. S7A, B.

**Fig. 7:**
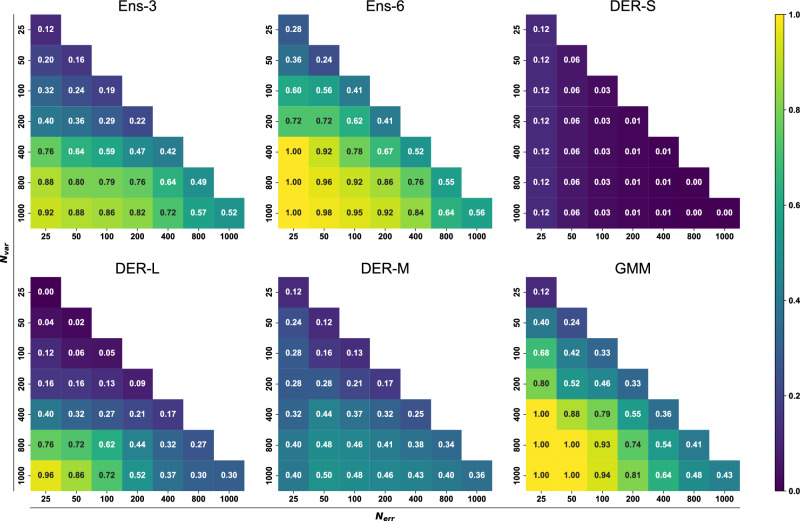
Bidimensional analysis of outlier-detection reliability for the different strategies. Given *N* structures with the highest error/variance, it is evaluated if they correspond to the *N* structures with the largest errors/variance. See Equation (12). The plot is colored according to the accuracy. Exact values of the accuracy are given for each combination in white.

Next, a 2-dimensional analysis involving different numbers of structures with large errors and different numbers of high-variance structures was carried out. Fig. 7 shows the probability of finding *N*_err_ structures with large error among the *N*_var_ structures with large variance for each method. As an example, for Ens-3, the lower left corner reports a probability of 0.92 for finding the *N*_err_ = 25 structures with large error among the *N*_var_ = 1000 structures with large variance. Increasing *N*_err_ to 1000 reduces this probability to 0.52. This row corresponds to the data reported in Fig. 6. More generally, the *N*_var_ can now be reduced from 1000 to 25, and the probability of finding corresponding large error predictions is reported in the full triangle. Light and dark colors correspond to high and low probabilities, respectively. In practice one wants to keep *N*_var_ small and increase the probability to find a maximum of *N*_err_ structures. From this perspective, the best-performing model is GMM.

With Ens-3 as the reference, Ens-6 and GMM perform slightly better overall, whereas DER-L is comparable for small *N*_err_ and large *N*_var_. As *N*_var_ decreases to 400 samples and below the reliability of DER-L drops drastically. DER-M performs inferior to DER-L for small *N*_err_ and large *N*_var_ but maintains a success rate of 0.2 to 0.4 for most values of *N*_err_ and *N*_var_. Finally, DER-S has the lowest success rate throughout except for *N*_err_ = *N*_var_ = 25 for which it performs better than DER-L. Complementary to the analysis presented here, other metrics such as the true positive rate, positive predictive values, false positives rate, and false negative rate were evaluated to quantify the reliability over the range of squared errors and variance (See SI for complementary discussion and Figs. S8–19). These analyses confirm the high reliability of ensemble models. In contrast, the worst performer is DER-S, with a low probability of detecting outliers. Finally, the calibration of the models was also evaluated by the construction of Root Mean-Squared Error v. Root MV diagrams. The ensuing calibration curves (See Fig. S20) indicate that none of the models is “well calibrated” which is based on the assumption that RMSE and RMV are linearly related, though. This may, however, not be the case for NN-trained PESs or chemical models in general, as was found recently^31^.

### Inside-outside distribution

**Fig. 8:**
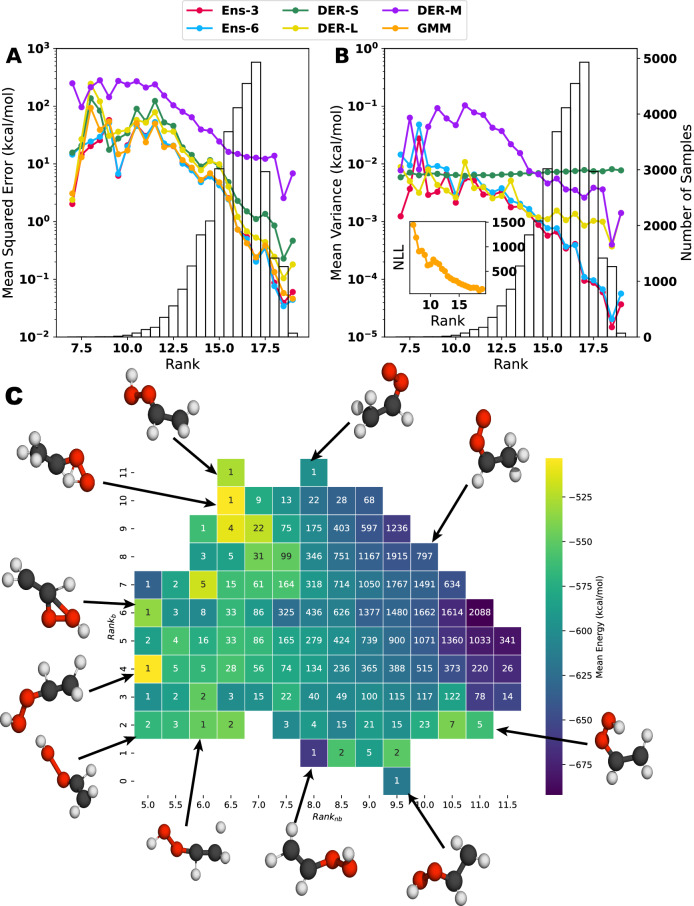
Relationships between the rank and mean squared error, mean variance, and structures. Evolution of the mean squared error (**A**) and the mean variance (**B**) concerning the rank of each structure in the test set. The bar plot (background) shows the number of structures with a particular rank. A large rank–value indicates that more degrees of freedom are covered by the training data and vice versa. The *y* axis is displayed in logarithmic scale to highlight the difference in the values of MSE or MV for the different rank values. Notice that for the Gaussian mixture model, the negative log-likelihood is used to estimate the uncertainty. The inset on the right panel shows how the mean NLL changes concerning the defined rank. **C** shows the 2d-map representation of the rank for bonded and non-bonded separations. Representative structures of different combinations are shown around the map.

A deeper understanding of the origin of the variances and the prediction error can be obtained by considering the distribution of structural features (atom distances) in the training and testing data sets, and to relate them to predicted properties. Following the procedure described in Section 2.3, a score (the *rank*) for each molecule in the test set was calculated. The results in Fig. 8 are combined with a histogram of the number of molecules with a given rank. The *rank*, see Equations (13) and (14), is interpreted as the degree to which a sample can be considered in or out of the distribution of atom separations covered by the training set: a high *rank* implies that more degrees of freedom (DOF) can be found in the training data. Thus, it is “in distribution” (ID), while a low *rank* indicates that the sample has more DOFs farther away from the distribution and is “out of distribution” (OOD). The black histogram in Fig. 8 shows that most samples have *rank* > 14 and are ID to some extent, with a most probable value *rank* = 17.

Figure 8 A, B indicate that *rank* and MSE or MV (colored lines) are related. Similarly, the distribution of samples with a given *rank* also impacts MSE and MV, see black histograms. For the MSE (Fig. 8A), all models except for DER-M behave similarly overall. Up to *rank* ~12, the MSE varies between $\sim\!\! 1$ and ~100 kcal/mol, and for *rank* >12 the MSE decays monotonically well below 1 kcal/mol for all models except for DER-M. For DER-M, the behavior is not fundamentally different, but the magnitude of the MSE is considerably increased. The MV in Fig. 8B follows the behavior of the MSE for Ens-3, Ens-6, DER-M, and GMM. For DER-L, the decay of the MV with increasing *rank* is less pronounced, whereas for DER-S MV ~0.1 kcal/mol throughout. One reason for the decay of MSE and MV with increasing *rank* is the larger number of samples for given *rank*, *P*(*rank*), see black histograms Fig. S21. What distinguishes DER-M from the other five methods is the fact that the achievable MSE remains considerably larger for most rank-values.

The relationship between *rank* and MSE/MV can also be considered individually for bonded and non-bonded separations; see Figs. S22–24. Overall, the results from Fig. 8A are replicated, but the relationship between *P*(*rank*) and the MSE is yet more pronounced for bonded terms. For small sample sizes, the MSE is large, and vice versa. Unexpectedly, for the non-bonded separations, the behavior for all models except for DER-M differs: For the lowest, sparsely populated *rank*—values the MSE increases with increasing *P*(*rank*) up to *rank* = 6.5, after which the MSE decreases monotonically. The MV, on the other hand, behaves as expected. It is noted that the MV for DER-S bonded and non-bonded separations is almost constant, irrespective of *P*(*rank*).

Values for bonded and non-bonded *rank* can also be analyzed in a 2-dimensional map, see Fig. 8C, which reports the average total energy and can be regarded as an abstract rendering of the PES. Low-energy structures correspond to the *syn-*Criegee and VHP basins, followed by structures representative of the TS between the reactant and product, and finally, higher-lying structures dominated by larger distortions. The majority of points (93%, white numbers in Fig. 8C) is for 8 ≤ *rank*_nb_ ≤ 11.5 and 4 ≤ *rank*_b_ ≤ 9. These structures cover an energy range from –700 to –300 kcal/mol with the lowest energy structures featuring *rank*_nb_ ≥ 11.0 and *rank*_b_ ≥ 5.0. Hence, these are comparatively “open” structures, characteristic of an elongated molecule such as the one considered here. A separate analysis for the relationship between MSE and MV for bonded and non-bonded *rank* is provided in Figs. S23 and S24 together with a more detailed discussion. This analysis relates to that reported in Fig. 8C.

The preceding analysis showed that a simple ranking such as the one presented here can highlight the effect of the differences between training and test distribution on the prediction and the uncertainty estimation. It must be mentioned that the *rank*—metric can be used as a proxy for how structure and error are related. However, further analysis is required to complement these results because averaging effects can play an important role. Yet, for improving reactive ML-PESs it is notable that samples with larger *rank* feature lower average error and vice versa. It is also found that coverage of the non-bonded distances for predicting energies and uncertainties can be rather informative. This contrasts with the usual focus on sufficiently covering the range of chemical bonds when conceiving data sets for training ML-PESs.

## Discussion

The present work analyzed quantitatively to what extent three different UQ methods—ensembles, DER, and GMM—are capable to detect outliers in samples from which full-dimensional reactive PESs can be trained. The system investigated for this was one of the criegee intermediates *syn-*Criegee, CH_3_CHOO.

**Fig. 9:**
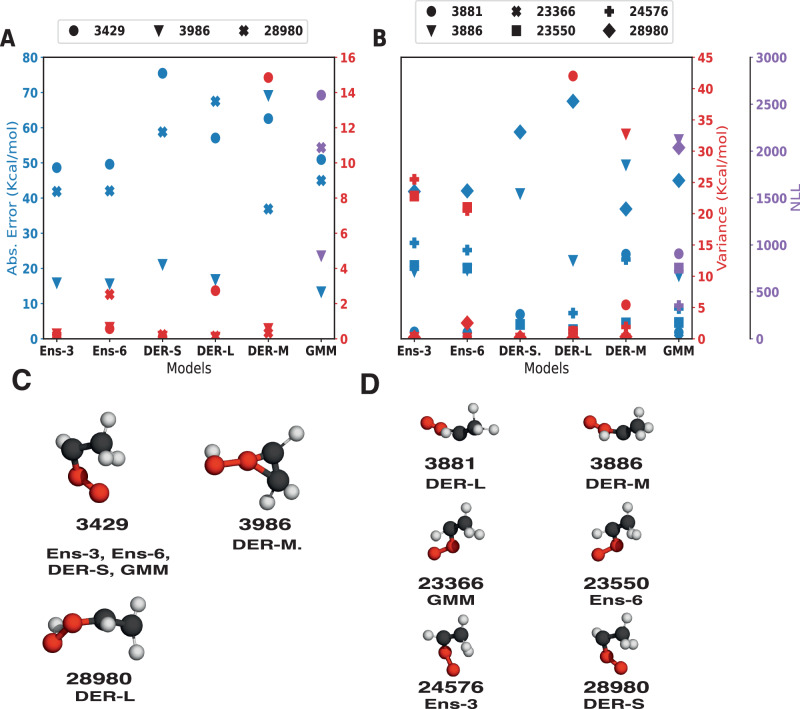
Extreme values in prediction. **A** shows the values of the absolute error (blue) and variance (red) or NLL (purple) for each of the samples identified to have the largest error and its corresponding index. Molecular structures are shown in **C** with their corresponding index and the model for which the structure is identified to have the largest error. **B** is similar to **A** but for the structures identified to have the largest variance. The corresponding structures are shown in **D**.

From an electronic structure perspective, criegee intermediates are known to be challenging because they feature Multireference (MR) effects^21,41^. This can also be demonstrated from the present data and even be linked to the quality of the prediction and the MV. For this, molecular structures with the largest absolute errors (Fig. 9A) and with the largest uncertainty (Fig. 9B) for each of the models were determined. Generally, the largest errors arise either for deformed *(syn)*-Criegee or VHP structures, whereas structures with the largest variance are predominantly perturbed *(syn)*-Criegee structures except for GMM, which identifies one structure closer to the TS. Interestingly, none of the models assigns the largest uncertainty to the structure with the largest error. In all cases, the magnitude of the error is larger than the predicted variance. On the other hand, for structures with large variance, the errors are on the same scale for ensembles and DER-M, whereas they are almost constant for DER-S. Contrary to this, DER-L overestimates the uncertainty by one order of magnitude.

Structure #3429 (see Fig. 9C) with the largest error is the same for four out of the six models. The remaining two models also show a large error for this structure, indicating that this structure is, in general, difficult to predict. Surprisingly, structure #3429 is predicted to have a large uncertainty for the models that do not identify it with the largest error (DER-M and DER-L), while the other four identify it with smaller uncertainty. Structure #3986 is the most difficult to predict with DER-M, while for the other models, it is better predicted with a difference between predictions of ≈50 kcal/mol. The GMM model assigns it a large uncertainty while the other models give it values in the same range as the predicted structure #3986. Lastly, structure #28980 features the largest error for DER-L but in the same magnitude as the other models except for DER-M. Regarding the uncertainty, Ens-6 identifies #28980 with a large uncertainty, while the other models attribute a small value to it. It is also found that Ens-3, Ens-6, DER-S, and GMM identify structures (e.g., #23366, #23550, #24576, #28980) that resemble those with the largest error; however, the error for these four structures is not large; see SI for a discussion.

One possible reason for the difficulties in predicting energies for particular geometrical arrangements concerns the MR character of its electronic structure. To prove this, the *T*_1_^42^ and *D*_1_^43^ diagnostic coefficients were determined, see Table S6. All structures with large errors clearly display MR characters, which are not captured from the single reference MP2 reference data used in the present work. Interestingly, the uncertainty prediction of the models appears to be related to the MR effects as well (Table S7) because the molecules identified with large variance also have large values of *T*_1_ and *D*_1_ diagnostic. These findings are also consistent with earlier work on acetaldehyde^44^.

From the present analysis, ensemble models emerge as a viable route for outlier detection. The capability of the modified DER models is considerably improved over DER-S, which is largely unsuitable for this task. On the other hand, DER-L is able to detect extreme cases with almost the same quality as the ensemble models thanks to the modifications of the loss function (*c.f*. Equation (6)). However, this capability decays rapidly with the number of required samples *N*_err_. Finally, DER-M has a constant probability of detecting outliers regardless of the number of samples considered. This is an interesting behavior because it implies a strong correlation between the error in prediction and the variance. Unfortunately, the probability of detecting outliers for DER-M is ~40% throughout. The last model, GMM, showed an intermediate performance between ensembles and DER. However, the NLL as the uncertainty measure is only qualitative and can not be used directly to estimate the error. Nevertheless, it performed well in detecting outliers with good reliability that decay at the same rate as ensemble models.

The fundamental insights gained from the present work are as follows. It is possible to carry out meaningful outlier detection for reactive PESs with the most successful approaches reaching 50% detection quality for a pool of 1000 structures with the highest uncertainty. Two new formulations of DER, DER-M, and DER-L, were presented and evaluated. The most promising among the approaches tested here are ensemble methods and DER-L, and it is found that Ens-6 and GMM yield consistent results overall. Large values of the *rank* metric (a geometry-based descriptor) were found to correlate with large average prediction errors suggesting that rapid-to-evaluate geometrical criteria may be a useful complement to detect outliers and to improve a given training set. A related structure-based procedure was successfully used for choosing structures best suited for transfer learning PESs for a specific process^45^. Potential future developments and improvements concern additional modifications to the loss function (scaled-by-variance^39^, *post-hoc* recalibration of the uncertainty using isotonic regression^46^) and exploring methods independent of the underlying statistics (such as Gaussian distribution of the data in DER) including conformal prediction methods^47,48^.

In the present work, a robust and validated PES from large-scale MD simulations^21,49^ was available a priori. This is an unusual situation: in real-world applications, a (reactive) PES needs to be constructed by starting from an initial sampling (i.e., MD, Normal Mode Sampling, etc., or combinations thereof), followed by refinement and augmentation of the data set based on active learning strategies^50^. For the second step, the use of UQ methods, together with heuristic criteria such as the *rank* considered here to rapidly assess whether new structures are likely to be “outliers” and, therefore, should be included in the training set is of critical relevance. Among the methods evaluated here, ensembles and the GMM perform best. However, for a fair and unbiased appreciation of the methods explored in the present work and their use in different applications, further studies are required.

## Methods

This section describes the ab initio reference data, the approaches to quantify uncertainty and further analyses. For the ensemble and DER models, the variance is used for UQ, whereas the negative log-likelihood (NLL) is used for the GMM. The “error” is the difference between the reference value of a property and the predicted value of that property with a given model whereas the “variance” is defined as the expected value for the squared difference between the predicted value and the mean value of the model. Finally, uncertainty is considered as the degree of confidence in the prediction made by a given model. Uncertainty is related to the lack of knowledge or the model’s limitations to describe a system^51^. In the text, “uncertainty” and “variance” are used synonymously, whereby a small variance value corresponds to a smaller uncertainty and a higher confidence in the prediction and vice versa. The models are characterized in terms of the mean squared error (MSE), the mean absolute error (MAE) and the MV.

### Data sets

The main ingredient for generating ML-PESs is reference electronic structure data to train the models on. Here, the H-transfer reaction from (*syn*)-Criegee to vinyl hydroxyperoxide (VHP) serves as a benchmark system (see Fig. 1) and reference data at the MP2/aug-cc-pVTZ level of theory is available from previous work^49^. From a total of 37,399 structures covering the H-transfer reaction for the *syn-*Criegee ↔ TS ↔ VHP reaction, ~10% were extracted semi-randomly (every 10th), and structures with very large energies (>400 kcal/mol above the minimum) are excluded. A total of 3706 data points were used for obtaining a first-generation ML-PES (see the energy distribution in Fig. S1). Multiple rounds of diffusion Monte Carlo simulations^52^ and adaptive sampling^53^ were run to detect *holes* and under-sampled regions. The resulting final data set contains a total of 4305 structures (see the energy distribution in Fig. S2) and is used to train new ML-PESs that are finally used for uncertainty prediction. It is important to note that the training data set is not considered to be comprehensive. If, e.g., a global PES for dissociation dynamics (i.e., formation of vinoxy radical, etc) is sought after, additional sampling would be required. Nevertheless, the small data set can be used to obtain different ML-based models and covers the relevant part of the configurational space of the reactive process of interest (H-transfer), and their ability to quantify uncertainty can be tested on an extensive test set. The (unseen) test set contains a total of 33402 structures covering the (*syn*)-Criegee ↔ VHP reaction and the distribution of energies is shown in Fig. S3.

### Methods for Uncertainty Quantification

#### Ensembles

The ensemble method based on the Query-by-committee^54^ strategy is a frequently used and practical approach to uncertainty estimation. For this strategy, a “committee” of ${\mathcal{N}}$ models is trained. The uncertainty measure is obtained as the disagreement between the models (or within the committee/ensemble). If the predictions of the ensemble members agree closely, it can be assumed that the region on the PES is well described. For under-sampled regions, however, the predictions will diverge^27^. Since many equivalent solutions to the NN optimization exist due to the nonconvexity of the problem at hand, diversity in the models should aid in reducing the error and improving the uncertainty^55^. Among others, model diversity can be improved by training multiple NNs with random initialization, early stopping (diversity could be included by using models from different stages during training), or bootstrapping (the ensemble members are trained in different bootstrap samples—a subset of the original data set—rather than the entire data set diversifying the models based on the data). However, as NNs generally achieve better performance with larger amounts of data, there is some evidence that bootstrapping is less beneficial^56^. A commonly used uncertainty measure for the ensemble is the standard deviation given by^27^

1 $${\sigma }_{E}=\sqrt{\frac{1}{{\mathcal{N}}}\mathop{\sum }\limits_{n}^{{\mathcal{N}}}{\left({\tilde{E}}_{n}-\bar{E}\right)}^{2}}.$$

Here, ${\mathcal{N}}$ corresponds to the number of committee models, ${\tilde{E}}_{n}$ is the energy predicted by committee model *n* and $\bar{E}$ is the ensemble average.

PhysNet^57^ was chosen to learn a representation of the PES. A total of 6 models were trained to generate an ensemble. All models share the same architecture and hyperparameters. In the present work, model diversity was ensured by a combination of random initialization of the learnable parameters following the Glorot initialization scheme^58^ (see Reference^57^ for details) and shuffling of the training/validation data. The latter is related to bootstrapping but does not reduce the training set size. From a total of six models, models 1/2, 3/4, and 5/6 were trained on exactly the same data and differed only in the initialization. The 4305 data points were split into training/validation sets according to 80/20%. The PhysNet models were trained on energies, forces, and dipole moments; details regarding PhysNet training are given below. Query-by-committee was performed with an ensemble of 6 models (Ens-6) and 3 models (Ens-3, models 1, 3, 5).

#### Deep evidential regression

The present work employs a modified architecture^31^ of PhysNet to predict energies and uncertainties based on DER. DER assumes that the energies are Gaussian-distributed $P(E)={\mathcal{N}}(\mu ,{\sigma }^{2})$. The prior distribution is a normal-inverse gamma (NIG), described by four values (*γ*, *ν*, *α*, *β*)^30^. The total loss function ${\mathcal{L}}$ includes the NLL, ${{\mathcal{L}}}^{NLL}(x)$, which is regularized by the *λ* − scaled MSE, ${{\mathcal{L}}}^{R}(x)$, that minimizes the evidence of incorrect predictions together with energies, forces, charges, and dipole moments for all structures in the training set

2 $$\begin{array}{l}{\mathcal{L}}={{\mathcal{L}}}^{NLL}({E}_{{\rm{ref}}},{E}_{{\rm{pred}}})+\lambda ({{\mathcal{L}}}^{R}({E}_{{\rm{ref}}},{E}_{{\rm{pred}}})-\varepsilon )+{W}_{F}| {F}_{{\rm{ref}}}-{F}_{{\rm{pred}}}| \\\qquad +{W}_{Q}\left\vert {Q}_{{\rm{ref}}}-{Q}_{{\rm{pred}}}\right\vert +{W}_{D}\left\vert {D}_{{\rm{ref}}}-{D}_{{\rm{pred}}}\right\vert .\end{array}$$

The NN is trained to minimize the difference between the NIG distribution and *p*(*E*). The values of the hyperparameters were *W*_*F*_ = 52.9177 Å/eV, *W*_*Q*_ = 14.3996 e^−1^, and *W*_*D*_ = 27.2113 *D*^−1^, respectively^57^, and *λ* = 0.15 and *ε* = 10^−4^ throughout. Note that the forces and dipole moments were calculated as in the original version of PhysNet. As a consequence, the variance of the forces can not be obtained because the derivative of the variance is the covariance matrix between energy and forces^59^. This model is referred to as DER-simple (DER-S).

#### Modified deep evidential regression

The effectiveness in predicting uncertainties by DER-S has been recently questioned^37,60^. Firstly, minimizing a loss function similar to Equation (2) is insufficient to uniquely determine the parameters of the NIG distribution because ${{\mathcal{L}}}^{NLL}({E}_{{\rm{ref}}},{E}_{{\rm{pred}}})$ is optimized independently of the data^37^. This leads to large uncertainty in poorly sampled regions. Secondly, it was shown that optimizing ${{\mathcal{L}}}^{NLL}({E}_{{\rm{ref}}},{E}_{{\rm{pred}}})$ is insufficient to obtain faithful predictions. Adding the term $\lambda ({{\mathcal{L}}}^{R}({E}_{{\rm{ref}}},{E}_{{\rm{pred}}})-\varepsilon )$ as a regularizer addresses this problem but can lead to a gradient conflict between the two terms^60^.

Two modifications to DER-S were considered. First, the multivariate generalization, DER-M, following the work of Meinert and Lavin^61^ was implemented. In DER-M, the NIG is replaced by a Normal-Inverse Wishart distribution, which is the multidimensional generalization of the NIG distribution to predict a multidimensional distribution of energies (*E*) and charges (*Q*). The loss function for DER-M is

3 $$\begin{array}{lll}{\mathcal{L}}\,=\,\log \left(\frac{\nu +1}{\nu -1}\right)-\nu \mathop{\sum}\limits _{j}{\ell }_{j}+\frac{\nu +1}{2}\log \left(\det \left({\bf{L}}{{\bf{L}}}^{\top }+\frac{1}{1+\nu }{\bf{Y}}\cdot {{\bf{Y}}}^{\top }\right)\right)\\\qquad\quad+\,{W}_{F}\left\vert {F}_{{\rm{pred}}}-{F}_{{\rm{ref}}}\right\vert +{W}_{D}\left\vert {D}_{{\rm{pred}}}-{D}_{{\rm{ref}}}\right\vert \end{array}$$

where ${\bf{Y}}={[{E}_{{\rm{ref}}},{Q}_{{\rm{r}}ef}]}^{\top }-{[{\mu }_{0},{\mu }_{1}]}^{\top }$. *μ*_0_ is the predicted energy (*E*_pred_) and *μ*_1_ the respective predicted total charge (*Q*_pred_). Then, the model output contains six values: the objective values (*E*_pred_, *Q*_p*red*_), the corresponding parameters of the covariance matrix **L,** $\overrightarrow{l}=diag({\bf{L}})$, and a parameter *ν*.

The outputs are constructed to be part of the covariance matrix **L** defined as

$${({\bf{L}})}_{ij}=\left\{\begin{array}{ll}{\rm{SoftPlus}}({\ell }_{i})+\epsilon &{\rm{If}}\,i=j\\ {l}_{ij}+\epsilon &{\rm{If}}\,i \,>\, j\\ 0&{\rm{else}}\end{array}\right.$$

Here, *ℓ*_*ij*_ are the outputs of the last layer (*E*_p*red*_, *Q*_pred_) of the modified PhysNet model. It must be mentioned that **L** is a lower triangular matrix. A difference between the original formulation of Meinert and Lavin^61^ and the one presented here is that the exponential function for the covariance matrix is replaced with the SoftPlus activation. Additionally, *ϵ* = 1 × 10^−6^ is added to each of the outputs of the last layer as a regularizer. These modifications avoid numerical instabilities and/or singularities during training.

The parameter *ν* corresponds to the number of DOF of the distribution^62^, and it is also an output of the PhysNet model. Meinert and Lavin^61^ relate *ν* to the number of virtual measurements of the variance. The value of *ν* is constrained to *ν* ∈ [3, 13]; the lower boundary corresponds to the requirement that *ν* > *n* + 1, where *n* is the number of predicted quantities. The upper boundary is *ν* < 13 because it is empirically known that for *ν* ≥ 13 the resulting distribution is indistinguishable from a normal distribution^63^. Then, the expression for *ν* is:

$$\nu =10\left(\frac{\tanh (x)+1}{2}\right)+3$$

The aleatoric (data) and epistemic (knowledge) uncertainty of the multidimensional model are obtained from

4 $${\mathbb{E}}[{\sigma }^{2}]=\frac{\nu }{\nu -3}{\bf{L}}{{\bf{L}}}^{\top }$$

5 $$Var[\mu ]=\frac{{\mathbb{E}}[{\sigma }^{2}]}{\nu }$$

For the second modified architecture, a Lipschitz-modified loss function ${{\mathcal{L}}}^{Lips}$ was used^60^ as a complementary regularization to the NLL loss

6 $$\begin{array}{r}{\mathcal{L}}={{\mathcal{L}}}^{NLL}({E}_{{\rm{ref}}},{E}_{{\rm{pred}}})+\lambda ({{\mathcal{L}}}^{R}({E}_{{\rm{ref}}},{E}_{{\rm{pred}}})-\varepsilon )+{{\mathcal{L}}}^{Lips.}({E}_{{\rm{ref}}},{E}_{{\rm{pred}}})\\ +{W}_{F}\left\vert {F}_{{\rm{ref}}}-{F}_{{\rm{pred}}}\right\vert +{W}_{Q}\left\vert {Q}_{{\rm{ref}}}-{Q}_{{\rm{pred}}}\right\vert +{W}_{D}\left\vert {D}_{{\rm{r}}ef}-{D}_{{\rm{pred}}}\right\vert \end{array}$$

Here, ${{\mathcal{L}}}^{Lips.}({E}_{{\rm{ref}}},{E}_{{\rm{pred}}})$ is defined as

7 $${{\mathcal{L}}}^{Lips.}({E}_{{\rm{ref}}},{E}_{{\rm{pred}}})=\left\{\begin{array}{ll}{({E}_{{\rm{ref}}}-{E}_{{\rm{pred}}})}^{2}&{\rm{If}}\,{\lambda }^{2} < {U}_{\nu ,\alpha }\\ 2\sqrt{{U}_{\nu ,\alpha }}| {E}_{{\rm{ref}}}-{E}_{{\rm{pred}}}| -{U}_{\nu ,\alpha }&{\rm{If}}\,{\lambda }^{2}\ge {U}_{\nu ,\alpha }\end{array}\right.$$

where ${\lambda }^{2}={({E}_{{\rm{ref}}}-{E}_{{\rm{pred}}})}^{2}$ and *U*_*α*,*ν*_ are the derivatives of ${{\mathcal{L}}}^{NLL}$ with respect to each variable

8 $$\left\{\begin{array}{l}{U}_{\nu }=\frac{\beta (\nu +1)}{\alpha \nu }\\ {U}_{\alpha }=\frac{2\beta (1+\nu )}{\nu }\left[\exp (\Psi (\alpha +1/2)-\Psi (\alpha ))-1\right]\end{array}\right.$$

and *Ψ*( ⋅ ) is the digamma function. This model is referred to as DER-L. For training DER-M and DER-L, the weights for forces, dipoles, and charges were the same as for DER-S.

#### Gaussian mixtures models

A third alternative to quantify the uncertainty is the so-called GMM. This method is convenient for representing—typically—multimodal distributions in terms of a combination of simpler distributions, such as multidimensional Gaussians^64^

9 $${\mathcal{N}}(x| {\mu }_{i},{\Sigma }_{i})=\frac{1}{{(2\pi )}^{D/2}| {\Sigma }_{i}{| }^{1/2}}\exp \left(-\frac{1}{2}{(x-{\mu }_{i})}^{\top }{\Sigma }_{i}^{-1}(x-{\mu }_{i})\right)$$

Here, *μ*_*i*_ is a *N*-dimensional mean vector and *Σ*_*i*_ is the *N* × *N*-dimensional covariance matrix. The distribution of data, here the distribution of molecular features, *x*, given parameters *θ* can be represented as a weighted sum of *N*-Gaussians:

10 $$p(x| \theta )=\mathop{\sum }\limits_{i=1}^{N}{\omega }_{i}{\mathcal{N}}(x| {\mu }_{i},{\Sigma }_{i})$$

with mixing coefficients *ω*_*i*_ obeying^65^$\mathop{\sum }\nolimits_{i = 1}^{N}{\omega }_{i}=1$ and 0 ≤ *ω*_*i*_ ≤ 1. The *ω*_*i*_ coefficients are the prior probability for the *i*th-component.

Following the work of Zhu et al.^66^, the parameters of Equation (10) (*θ* = {*ω*_*i*_, *μ*_*i*_, *Σ*_*i*_}) to construct the GMM were obtained from the molecular features of the last layer of a trained PhysNet model, *i.e*. one of the ensemble members. The distribution of molecular features from the training set is used to acquire the values of *θ*. The initial *μ*_*i*_ values were determined from k-means clustering. To each Gaussian *i* in the GMM model, a covariance matrix *Σ*_*i*_ is assigned. The number of Gaussian functions required was determined by using the Bayesian information criterion (BIC) and was *N* = 37. Finally, the fitted model was evaluated by using the NLL of the molecular feature vector as:

11 $$NLL(p(x| X))=-\ln \left(\mathop{\sum }\limits_{i = 1}^{N}{\omega }_{i}{\mathcal{N}}(x| {\mu }_{i},{\Sigma }_{i})\right)$$

Here, *p*(*x*∣*X*) is the conditional probability of a molecular feature vector *x* with respect to the distribution of feature vectors in the training data set *X*. The value of NLL is used as a measure of the uncertainty prediction, whereby smaller NLL values indicate good agreement. The “detour” involving the feature vectors is a disadvantage over the other methods studied here because it is not possible to relate the predicted energy with the corresponding uncertainty directly.

### Set up of the NN training

The NN model used in this work is PhysNet^57^. The original version in Tensorflow was used for the ensemble method, while the Pytorch version was employed for DER. Five modules were used in both cases, each with two residual atomic modules and three residual interaction modules. The output of it was pooled into one residual output model. The number of radial basis functions was kept at 64, and the dimensionality of the feature space was 128. A batch size of 32 and a learning rate of 0.001 were used for training. An exponential learning rate scheduler with a decay factor of 0.1 every 1000 steps and the ADAM optimizer^67^ with a weight decay of 0.1 were employed. An exponential moving average for all the parameters was used to prevent overfitting. A validation step was performed every five epochs.

### Analysis

#### Outlier detection

In this work, the quality of outlier detection is measured by considering whether a number *N*_error_ of samples with the largest error Δ*E* = *E*_ref_−*E*_p*red*_ can be found within the *N*_var_ structures with the highest variance (or NLL in the case of GMM) as determined from UQ. Therefore, the accuracy for detecting outliers is defined as:

12 $$Acc=\frac{n({N}_{{\rm{error}}}\cap {N}_{{\rm{var}}})}{{N}_{{\rm{var}}}}$$

Here, *n*( ⋅ ) is the cardinality of the intersection between the set of samples with the largest errors and the set with the largest variances. Complementary to this, a classification analysis of prediction over error and predicted variance was performed.

#### Inside–outside distribution

The definition of inside-outside distribution is a controversial topic in the ML literature^68^. Here, the natural definition of statistical learning theory is used^69^: Assume a training data distribution *p*_train_(*x*) and a test data distribution *q*_test_(*x*) are given; a point *x*_*i*_ is defined as out-of-distribution if

$${q}_{{\rm{test}}}({x}_{i})\,\ne\, {p}_{{\rm{train}}}({x}_{i}).$$

In other words: if for each atom-atom separation *r*_*i*_ the training distribution is bounded by $[{r}_{i}^{\min },{r}_{i}^{\max }]$, the structure *r*_*i*_ is “in distribution” if ${r}_{i}^{\min }\le {r}_{i}\le {r}_{i}^{\max }$ for all *i*. This definition is “natural” within statistical learning theory. However, other possibilities based on an energy-based criteria^70,71^, score functions^72^, or nearest neighbors^73^ can also be used.

Determining whether a sample is in- or out-of distribution is a challenging task. In the present work, a metric based on the distribution of intermolecular distances is introduced. This metric, referred to as *rank*, is particularly suited to query PESs but may also be useful whenever the bonding pattern of molecules changes. The *rank* is based on the working hypothesis that if a distance in the test set can be found among the more common values in the distribution of distances used to train an ML model, the model must be able to predict it. Similar methods based on calculating the distance in the latent/descriptor space have been used for UQ^31,74^ and can be considered as a one-dimensional formulation of the GMM method. Different from these methods, the *rank* can be obtained from the structure alone, i.e., even before training the NN. In other words, it can be determined before constructing the training database.

The *rank* is determined as follows: First, all *R* = 28 intramolecular distances were computed. These distances were classified into “bonded” and “non-bonded” separations as follows: if a distance is smaller than the mean of the van der Waals radii of the two atoms concerned plus 20%, the value is considered “bonded"; otherwise it is non-bonded. The van der Waals radii used^75^ were 1.10 Å, 1.70 Å, and 1.52 Å, for H-, C-, and O-atoms. Next, the 28 distances were computed for all structures in the training data set to determine *p*_bond_(*r*) and *p*_no-bond_(*r*). Using these distributions, it was possible to query a given distance of the samples in the test data set to be inside (*Q*_5%_(*r*) < *r*_*i*_ < *Q*_95%_(*r*)) or outside (otherwise) the distribution *p*(*r*). Here *Q*_5%_(*r*) and *Q*_95%_(*r*) are the 5% and 95% quantile of *p*(*r*). Using this criterion, the contribution *χ*_*j*_(*r*_*i*_) of distance *r*_*i*_ for structure *j* is

13 $${\chi }_{j}({r}_{i})=\left\{\begin{array}{ll}1\quad &{r}_{i}\in {p}_{{\rm{bond}}}(r)\\ 0.5\quad &{r}_{i}\in {p}_{{\rm{no-bond}}}(r)\\ 0\quad &{r}_{i}\,\notin\, [{p}_{{\rm{bond}}}(r)\cap {p}_{{\rm{no-bond}}}(r)]\end{array}\right.$$

From this, *rank*_*j*_ for sample *j* was determined according to

14 $$ran{k}_{j}=\mathop{\sum }\limits_{i}^{R=28}{\chi }_{j}({r}_{i})$$

Using the mean of van der Waals radii to determine the *rank* is only one possibility. Alternative metrics based on covalent radii, bond orders, or electron densities may be considered and yield somewhat different results. Also, depending on how the scores are assigned in Eq. (13), results may vary. However, the influence of all these considerations can be explicitly tested and computation of the *rank* can be refined in future work.

#### Classification

Following the methodology presented by Kahle and Zipoli^76^, we classified the predictions obtained by the different models to determine if the predicted uncertainty can be used as a reliable estimation of the prediction error. In this case, the following classes were defined:

- True Positive (TP): *ε*_*i*_ > ε* and *σ*_*i*_ > σ*.
- False Positive (FP): *ε*_*i*_ < ε* and *σ*_*i*_ > σ*
- True Negative (TN): *ε*_*i*_ < ε* and *σ*_*i*_ < σ*.
- False Negative (FN): *ε*_*i*_ > ε* and *σ*_*i*_ < σ*.

As a difference from our previous approach^31^, we report the results when ε* = MSE and σ* = MV and also for different values of σ* and ε* to obtain decision boundaries for the relationship between variance and error. For the different values of σ* and ε*, common metrics of the overall performance were evaluated. In this work, we use the true positive rate (*R*_TP_) or *sensitivity*. This quantity is defined as^77^:

15 $${R}_{{\rm{TP}}}=\frac{{N}_{{\rm{TP}}}}{{N}_{{\rm{TP}}}+{N}_{{\rm{FN}}}}$$

Here, *N*_TP_ refers to the number of true positives, and *N*_FN_ to the number of false negative samples. A large sensitivity value indicates that the model is unlikely to relate large variance values with small errors (*c.f.* false negatives).

Complementary to Equation (15) is the positive predictive value (*P*_TP_) or *precision*:

16 $${P}_{{\rm{TP}}}=\frac{{N}_{{\rm{TP}}}}{{N}_{{\rm{TP}}}+{N}_{{\rm{FP}}}}$$

where all the previous quantities keep their meaning, and *N*_FP_ is the number of false positives. This quantity relates to how many of the samples predicted with high uncertainty correspond to a large error.

In addition, it is desirable to quantify how often the model misclassifies a prediction. This can be measured by the false positive rate, which measures how many samples are classified with large uncertainty but low error. This is defined as:

17 $${R}_{{\rm{FP}}}=\frac{{N}_{{\rm{FP}}}}{{N}_{{\rm{FP}}}+{N}_{{\rm{TN}}}}$$

The opposite case can be quantified by the false negative rate defined as:

18 $${R}_{{\rm{FN}}}=\frac{{N}_{{\rm{FN}}}}{{N}_{{\rm{TP}}}+{N}_{{\rm{FN}}}}$$

#### Evaluating the multi reference character of a structure

Determining if a single reference method adequately describes a molecular system is challenging. Therefore, several diagnostic metrics have been proposed to evaluate MR effects on the system. Among them is the *T*_1_ diagnostic^42,78^, which is the Euclidean norm of the single substitution amplitudes vector (*t*_1_) of the closed-shell couple-cluster single doubles (CCSD) wave function divided by the square root of the number of correlated electrons:

19 $${T}_{1}=\frac{\left\Vert {t}_{1}\right\Vert }{\sqrt{{N}_{{\rm{corr}}.{\rm{elec}}.}}}$$

A single reference method will perform correctly if the value of the *T*_1_ diagnostic^79^ is *T*_1_ < 0.02. Complementary, the *D*_1_ diagnostic^43^ is defined as the maximum Euclidean norm of the vectors formed by the product of the matrix ***S*** which elements ${s}_{1}^{2}$ are the single excitation amplitudes of the CCSD wave function. Then, the *D*_*i*_ diagnostic is defined as:

20 $${D}_{1}=\parallel\, {\boldsymbol{S}}\,| {| }_{2}=\mathop{\max }\limits_{| | x| {| }_{2}=1}\left(| | {\boldsymbol{S}}\overrightarrow{x}{\parallel }_{2}\right)$$

Here ${\boldsymbol{S}}\in {{\mathbb{R}}}^{o\times v}$ with *o* and *v* denoting the number of active occupied and active virtual orbitals, respectively. For *D*_1_ > 0.05 the molecule is dominated by dynamic correlation^79^. *T*_1_ and *D*_1_ are suggested to be used together because *T*_1_ represents an average value for the complete molecule, which might fail to indicate problems for small regions of the molecule. In those cases, *D*_1_ can be used as evidence if the molecule has regions that single reference methods can not adequately describe.

In this work, *T*_1_ and *D*_1_ diagnostics were determined for the structures identified with the largest error for each model tested and those with the largest uncertainty value. Then, each molecule was computed at the CCSD(T)-F12 level of theory with the aug-cc-pVTZ basis function with the MOLPRO suite^80^. Then, the values of *T*_1_ and *D*_1_ are reported on Table S6 for the molecules with large errors and Table S7 for those with large variance.

### Energy conservation

The energy conservation of the models was estimated by running MD simulations using the generated potentials within the atomic simulation environment^81^. *NVE* simulations were run using Verlet dynamics. The initial velocities were assigned to follow a Maxwell-Boltzmann distribution at 300 K. Each simulation was run from the *(syn)*-Criegee intermediate for 0.5 ns using a time step of 0.1 fs and the energies were saved every 1000 steps.

## Supplementary information

Supporting information

## Data Availability

The data supporting this research are available at https://github.com/MMunibas/outlier.

## Code availability

The code supporting this research is available at https://github.com/MMunibas/outlier.
