# Supplementary material for: Outlier-detection for reactive machine learned potential energy surfaces
Source: NPJ Comput Mater. 2025 Feb 15;11(1):33. doi: 10.1038/s41524-024-01473-6 (PMC11829830; doi:10.1038/s41524-024-01473-6)
Supplement: Supplementary file 1 — Supporting information [file 41524_2024_1473_MOESM1_ESM.pdf]

Supporting Information: Outlier-Detection for Reactive Machine Learned Potential Energy Surfaces

Luis Itza Vazquez-Salazar,[*,†,‡] Silvan Käser,[*,†,‡] and Markus Meuwly[*,†]

†*Department of Chemistry, University of Basel, Klingelbergstrasse 80 , CH-4056 Basel, Switzerland.*

‡*These authors contributed equally*

E-mail: luisitza.vazquezsalazar@unibas.ch; silvan.kaeser@unibas.ch; m.meuwly@unibas.ch

January 24, 2025

1 Supplementary Discussion

1.1 Characterization of the Trained PESs: Root Mean Square Displacement

Further to reporting the energetics of the equilibrium structures in the main manuscript, the Root Mean Squared Displacement (RMSD) between optimized geometries from the trained NN models and at the MP2 level are discussed, see Figure S4. Generally, the deviations between the obtained geometries and the reference structures are very small. However, some differences between the tested models can be highlighted. First, it is noticed that models that use DER have an RMSD two or three orders of magnitude larger than ensembles. Additionally, it is observed that the geometry of the TS is predicted more accurately than the *(syn)*-Criegee or VHP conformations. For the DER models, the geometries obtained with DER-S are the most accurate by approximately two orders of magnitude compared to the ones produced with their counterparts. On the other hand, structures obtained with DER-M have the largest RMSD among the models tested here. The last of the DER models tested, DER-L, produces constant RMSD for the different molecules. Finally, the results obtained with GMM are slightly lower quality than those from the ensemble models. This is expected because the GMM model is based on one of the ensemble members.

1.2 Outlier Detection: Metrics of Classification

Complementary to the reliability analysis in Figures 6 and 8 of the main manuscript, additional metrics are considered to evaluate the capabilities and limitations of the models used in this work. To this end, the true positive rate (sensitivity or TPR, Eq. 15), that quantifies

how many of the samples identified with a large variance also have a large error (*c.f.* true positives), and the positive predictive values (precision or PPV, Eq. 16) that measure how many of the samples with a large error are correctly labelled by the model were analyzed. This test was performed over different ranges of squared error and variance (or NLL for GMM), which can be used as confidence boundaries. Ideally, the model is expected to have large sensitivity and precision. Results for this analysis are shown in Figures S8-S13, which report a heatmap of TPR and PPV values using different thresholds for error or variance in the plot. Larger (desired) values are coloured blue, while small values are shown in red. The results indicate that Ens-6 and Ens-3 have high sensitivity for all error ranges at low variance values (Figure S8 and S9). Conversely, PPV values are high at all variance ranges for a small error cutoff. It is also observed that the confidence range for Ens-6 (Figure S9) is larger than for Ens-3 (Figure S8). Results for the DER models also have large TPR values at small uncertainty values (Figures S10, S11 and S12). On the contrary, the PPV coverage is almost null for DER-S (Figure S10) and DER-L (Figure S11), while DER-M has high values for all variance ranges with a small error threshold (Figure S12). Note, however, that the scales for squared error and variance differ by 2 to 3 orders of magnitude. Hence, the magnitude of the MSE and MV needs to be carefully inspected in addition to the colour code. Lastly, the TPR for GMM shows a good performance over a large range of NLL values, which implies the model correctly assigns uncertainty to errors in a larger range of uncertainty (Figure S13). On the other hand, PPV values are obtained for large values of NLL but low squared error threshold (Figure S13).

Finally, two more metrics to quantify the reliability over the range of squared errors and variance were evaluated. The first is the false positives rate (FPR, Eq. 17), also known as ”false alarm rate”, which measures how many of the samples identified with large variance do not correspond to a large error. Secondly, the false negative rate (FNR, Eq. 18) or ”miss rate” quantifies how many samples not identified with a large variance correspond

to a large error. For FPR and FNR small values (red) are desirable, whereas large values (blue) are undesirable. The results for both metrics are shown in Figures S14 to S19. For the ensemble models, FPR ~ 0 over the range evaluated (Figures S14 and S15), indicating a low probability of misclassifying samples, i.e. suitable for outlier detection. Complementary, the FNR values are small for small variance values (Figures S14 and S15 left), while the probability of missing a sample with a large error increases with the variance. The results for DER models show low values of FPR except for very small values of variance (Figures S16, S17, and S18 left). Regarding the results for the FNR, large values are obtained except for very small values of variance (Figure S16, S17, and S18 right). Finally, the GMM model has large values of FPR at low values of NLL (Figure S19 left) while the values of FNR are low in a large region but decay rapidly at large values of NLL (Figure S19 right). These results suggest that Ens-6 is the best model for detecting outliers with high TPR, and PPV complemented with a low FPR and FNR. On the contrary, the worst model is DER-S, which has a low probability of identifying outliers.

Lastly, different metrics for the evaluation of the calibration of the models were evaluated (See Figure S20). A limitation of these methods is that the GMM method can not be evaluated as those require a direct estimation of the variance. The first metric evaluated for the calibration of the models is the RMSE v. RMV diagram.[S1] This diagram indicates that a model is well calibrated as the slope of the plot is close to 1. The results in Figure S20 A) indicate that none of the models fulfils the requirement with slopes larger than one, indicating overconfidence in the predictions. However, in previous studies,[S2] it was found that a simple linear relationship between RMSE and RMV is difficult to achieve. The second metric of calibration evaluated is the construction of 'calibration diagrams' that report the frequency of correctly predicted values in each interval relative to the predicted fraction of points in that interval.[S2,S3] The results in Figure S20B indicate that the best performers are Ens-6 and DER-L. This is further confirmed by the value of the miscalibration area.

Nevertheless, the results must be taken with caution as it is known that this method presents several flaws, such as the obtention of a perfect calibration even if the output values for the variance of a model are independent of the observed error or if the prediction of uncertainty is degenerated.[S1]

1.3 Inside-Outside Distribution: Relationship between *rank* and MSE/MV for Bonded and Non-bonded Distances

The relationship between *rank* and the MSE/MV can also be analyzed separately for bonded and non-bonded separations and mapped on the 2d-representation as in Figure 8C, see Figures S23 and S24. Hence, the map itself remains, but the colouration changes. For the MSE, darker colours indicate a low error, whereas lighter colours indicate higher errors. The re-gions for high MSE remain the same for all six models considered: $5.0 \leq rank_{\mathrm{nb}} \leq 7.5$ and $2 \leq rank_{\mathrm{b}} \leq 5$, i.e. What changes, however, is the *maximum* MSE which is 9 kcal/mol for Ens-3 and Ens-6 and increases up to 40 kcal/mol for DER-M.

For the MV, Ens-3 and Ens-6 are on the same scale and differ l ittle. T he l argest variances for Ens-3 and Ens-6 are observed for similar ranks as for the MSE. On the other hand, DER-S, DER-M and DER-L are on rather different s cales r anging f rom 1 0^{-3} (DER-S) to ~ 0.1 kcal/mol (DER-M and DER-L). DER-S returns a uniform value for all values of $rank_{\mathrm{b}}$ and $rank_{\mathrm{nb}}$. For DER-L, the MV is larger for $5.0 \leq rank_{\mathrm{nb}} \leq 7.5$ and $0 \leq rank_{\mathrm{b}} \leq 9$, while DER-M displays large values for a wider region ($rank_{\mathrm{nb}} \leq 9.5$, $rank_{\mathrm{b}} \leq 8$). Finally, the magnitude of NLL for GMM can not be directly compared with the other five models, but NLL is large for $rank_{\mathrm{nb}} \leq 8$, $rank_{\mathrm{b}} \leq 8$.

1.4 Analysis of Structures with the Largest Variance

Structures with large variance are analyzed similarly to those with a large error; see "Discussion and Conclusion" and Figure 10 in the main manuscript. These samples present more considerable structural diversity for error and variance than the corresponding structures with the biggest values of error (Figures 10C and D). In the following, we will describe the error and variance for each structure following the enumeration of the samples shown in Figure 10D. Test structure #3881 is related to the largest uncertainty for DER-L; this is only replicated by DER-M and GMM, which also assigns it a large uncertainty. However, the energy prediction is accurate for most of the models evaluated except for DER-S. Next, structure #3886 has the largest uncertainty for DER-M, while none of the other models associates it with a large uncertainty value. Nevertheless, this structure is hard to predict for all of them, with errors between 50 and 20 kcal/mol. Continuing with our analysis, molecule #11467 is discussed. This sample is identified with the largest uncertainty value for the GMM model. Nonetheless, all models, even GMM, perform well in predicting this sample. Structures #23550 and #24576 are identified with the largest variance for the models Ens-6 and Ens-3, respectively. Both structures are similar, with a difference in the orientation of the carbon atom attached to the O-O in the *(syn)*-Criegee complex. Both samples show problems to be predicted by the ensemble models; however, it looks like models based on DER show fewer difficulties. Regarding the predicted uncertainty for #23550 and #24576, GMM assigns it a large uncertainty while the DER models assign it a low uncertainty. Last but not least is sample #28980, which is identified with the largest variance for the DER-S model. This sample is hard to predict for all models, being the hardest for DER-L, which yields the largest error for it. Regarding the uncertainty, it is noticed that for most of the models, with the exception of Ens-6, the predicted uncertainty is low. This analysis clearly shows that the prediction error is comparable for most of the analyzed models. However, detecting this error is not easy, as none of the extreme uncertainty values predicted are related to the extreme error.

2 Supplementary Tables

Table S1: Summary of the statistical metrics of the predictions of energy and forces for the models tested in this work. The first two columns correspond to the values for energies, while the last two columns are the values for forces. Units are kcal/mol for energies and (kcal/mol)·Å^{-1} for forces.

Model	MAE(E)	RMSE(E)	MAE(F)	RMSE(F)
Ens-3	0.44	1.80	1.54	11.98
Ens-6	0.43	1.79	1.48	11.47
DER-S	1.03	2.61	32.06	90.60
DER-L	0.69	2.35	31.79	90.09
DER-M	2.19	5.17	33.55	91.54
GMM	0.47	1.83	1.68	9.73

Table S2: Harmonic frequencies of *(syn)*-Criegee: *Ab initio* MP2 reference values are compared to the frequencies determined on the different PESs.

s-Cri.	MP2 Ref.	Ens-3	Ens-6	DER-S	DER-L	DER-M	GMM
1	224.2	225.9	222.8	251.7	170.8	218.2	223.8
2	304.0	298.8	297.2	337.0	273.3	479.0	300.0
3	481.5	476.2	475.8	440.0	460.6	518.8	475.7
4	698.5	691.6	691.2	679.2	687.9	686.6	691.2
5	745.3	738.2	738.1	710.9	761.6	750.6	738.8
6	939.6	928.3	928.6	919.0	924.2	951.9	927.9
7	996.4	998.7	998.3	1000.7	993.0	1010.8	998.8
8	1031.1	1035.1	1034.8	1018.6	1019.7	1067.4	1035.3
9	1130.3	1132.2	1132.0	1112.8	1118.6	1237.4	1132.4
10	1295.6	1286.9	1287.4	1305.4	1300.1	1328.2	1288.6
11	1397.6	1397.4	1397.4	1379.0	1390.9	1387.2	1397.1
12	1456.6	1451.3	1451.2	1403.4	1450.5	1441.0	1451.1
13	1474.2	1471.3	1471.2	1484.4	1486.9	1494.1	1471.2
14	1514.3	1513.1	1513.5	1541.3	1525.9	1540.5	1514.6
15	3047.8	3044.2	3045.1	3060.4	3030.3	2818.8	3046.7
16	3101.5	3088.9	3090.2	3148.6	3069.4	3085.0	3091.0
17	3207.3	3206.2	3206.5	3171.7	3198.7	3126.4	3210.1
18	3253.2	3255.9	3255.9	3186.7	3301.3	3253.7	3256.9
**MAE**	-	4.7	4.5	27.3	17.9	46.5	4.4

Table S3: Harmonic frequencies of transition state: *Ab initio* MP2 reference values are compared to the frequencies determined on the different PESs.

**TS**	**MP2 Ref.**	**Ens-3**	**Ens-6**	**DER-S**	**DER-L**	**DER-M**	**GMM**
1	518.0	517.4	517.4	494.4	506.0	453.4	517.4
2	533.0	528.5	528.5	541.3	524.2	502.2	528.4
3	745.3	744.9	744.9	715.7	721.0	686.0	744.9
4	770.9	768.6	768.6	765.7	748.2	766.5	768.6
5	857.7	853.7	853.7	845.5	846.0	833.7	853.8
6	969.9	964.0	964.0	929.0	932.1	973.2	964.0
7	1010.3	1007.4	1007.4	992.2	1000.4	1011.2	1007.4
8	1036.7	1033.2	1033.2	1030.7	1042.4	1063.8	1033.3
9	1223.2	1221.3	1221.3	1201.0	1220.8	1184.8	1221.3
10	1281.6	1281.2	1281.2	1272.0	1296.0	1250.9	1281.2
11	1360.3	1360.0	1360.1	1329.5	1382.1	1412.2	1360.0
12	1504.5	1503.3	1503.3	1466.9	1510.7	1555.6	1503.2
13	1557.9	1554.2	1554.2	1542.5	1564.2	1572.7	1554.2
14	1875.3	1866.3	1866.4	1795.1	1805.2	2021.0	1866.1
15	3116.3	3118.9	3118.8	3095.8	3071.0	3124.2	3118.7
16	3237.2	3236.3	3236.3	3215.1	3130.6	3235.1	3236.0
17	3251.9	3252.9	3252.9	3230.5	3159.4	3264.3	3252.8
i	1523.0	1518.3	1518.2	1574.3	1544.7	1331.7	1518.5
**MAE**	-	2.8	2.8	25.3	28.9	42.3	2.8

Table S4: Harmonic frequencies of VHP: *Ab initio* MP2 reference values are compared to the frequencies determined on the different PESs.

VHP	MP2 Ref.	Ens-3	Ens-6	DER-S	DER-L	DER-M	GMM
1	149.1	178.3	178.7	194.1	176.2	209.8	178.7
2	253.1	254.4	254.4	258	240.6	250.9	254.5
3	332.5	331.8	331.8	338.5	338.5	376.4	331.8
4	612.4	613.0	613.0	622.4	595.6	562.0	613.2
5	711.2	708.7	708.7	668.5	626.0	602.0	708.8
6	843.8	840.7	840.6	797.6	783.9	796.6	840.8
7	878.3	876.1	876.0	878.5	859.2	839.3	876.2
8	972.2	968.2	968.3	909.0	878.1	890.1	968.4
9	975.0	971.7	971.7	994.6	988.4	1030.6	971.9
10	1158.8	1156.3	1156.2	1152.8	1153.6	1130.2	1156.4
11	1319.1	1319.1	1319.1	1340.6	1372.8	1270.9	1319.1
12	1374.2	1372.6	1372.6	1350.4	1388.3	1325.9	1372.7
13	1428.7	1425.4	1425.4	1449.6	1417.4	1464.7	1425.4
14	1693.6	1691.6	1691.6	1704.9	1711.8	1689.2	1691.6
15	3216.3	3222.5	3222.4	3144.9	3178.9	3191.5	3222.3
16	3236.0	3235.3	3235.2	3178.8	3237.1	3299.5	3235.1
17	3330.0	3333.7	3333.8	3313.4	3289.2	3393.3	3333.4
18	3762.9	3759.0	3758.9	3716.0	3765.8	3821.4	3758.8
**MAE**	-	3.9	4.0	28.5	28.8	48.1	3.9

Table S5: Range of errors and variances/NLL values of the first 1000 samples with the largest values of error or variances for the different models tested in this work. Units are kcal/mol.

Model	Squared Error		Variance/NLL	
	Min	Max	Min	Max
Ens-3	0.388	103.164	0.075	25.562
Ens-6	0.354	107.326	0.094	21.051
DER-S	1.026	248.283	0.210	0.230
DER-L	0.715	198.678	0.102	42.130
DER-M	9.160	208.599	1.160	32.834
GMM	0.417	113.157	792.013	4096.360

Table S6: Diagnostics for assessing the multi-reference character of the structures identified with the largest error in the test dataset. These quantities are unitless. A value of $T_1 > 0.02$ indicates a multi-reference character, and $D_1 > 0.05$ points to dynamical multi-reference effects.[S4]

Molecule	T_1	D_1
3429	0.09	0.45
3986	0.05	0.23
28980	0.05	0.24

Table S7: Diagnostic metrics for the multi-reference character of the structures identified with the largest uncertainty in the test dataset. A value of $T_1 > 0.02$ indicates a multi-reference character. Complementary, $D_1 > 0.05$ indicates the presence of dynamical multi-reference effects.

Molecule	T_1	D_1
3881	0.07	0.25
3886	0.08	0.35
23366	0.04	0.19
23550	0.05	0.24
24576	0.07	0.36

3 Supplementary Figures

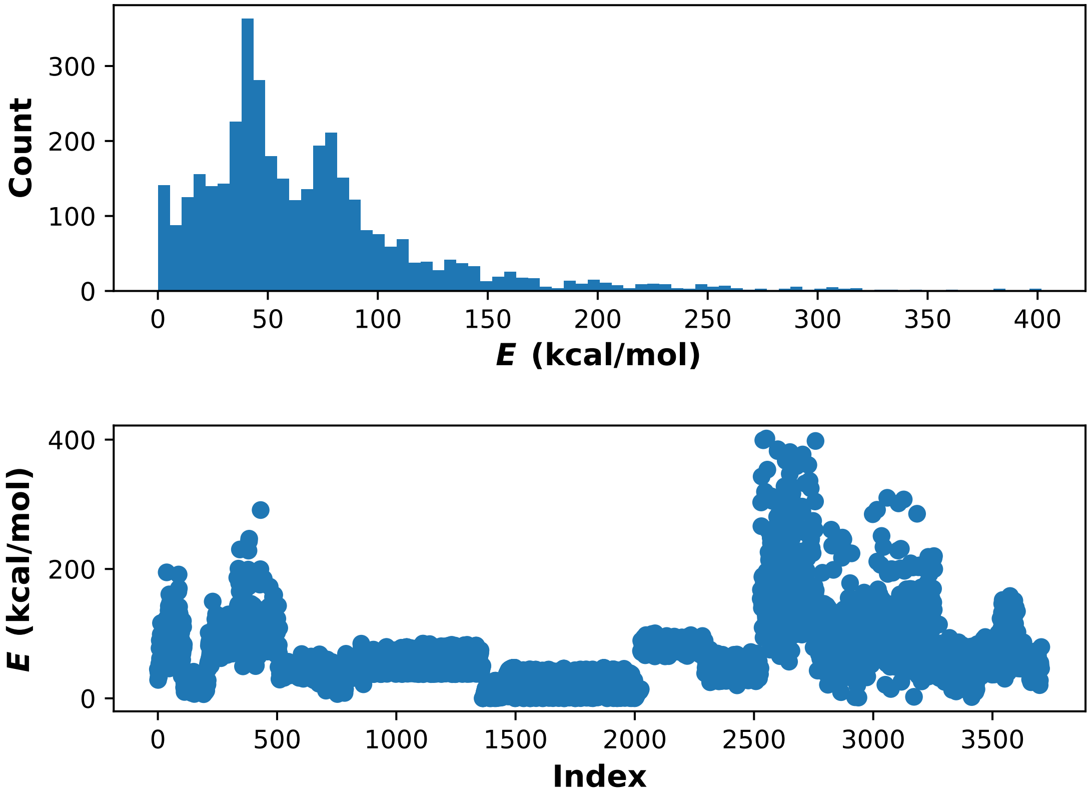

Figure S1: Energy distribution of the data set employed to train the first generation ML-PES.

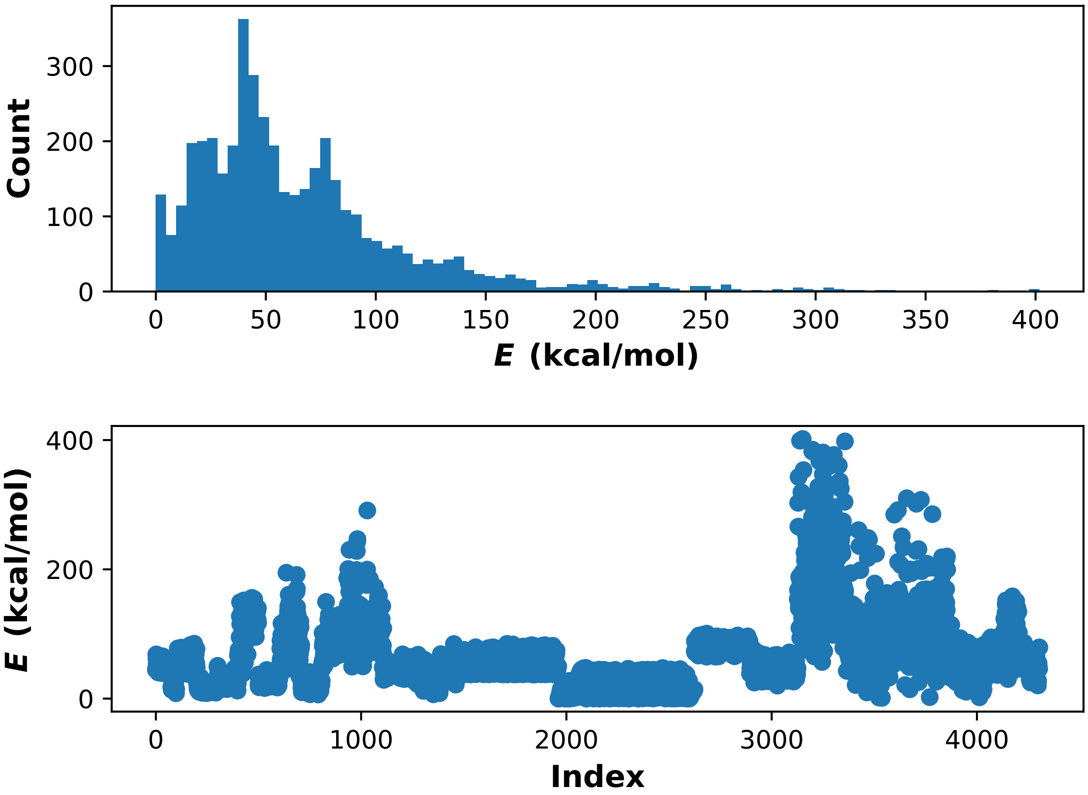

Figure S2: Energy distribution of the data set employed to train the final generation ML-PES.

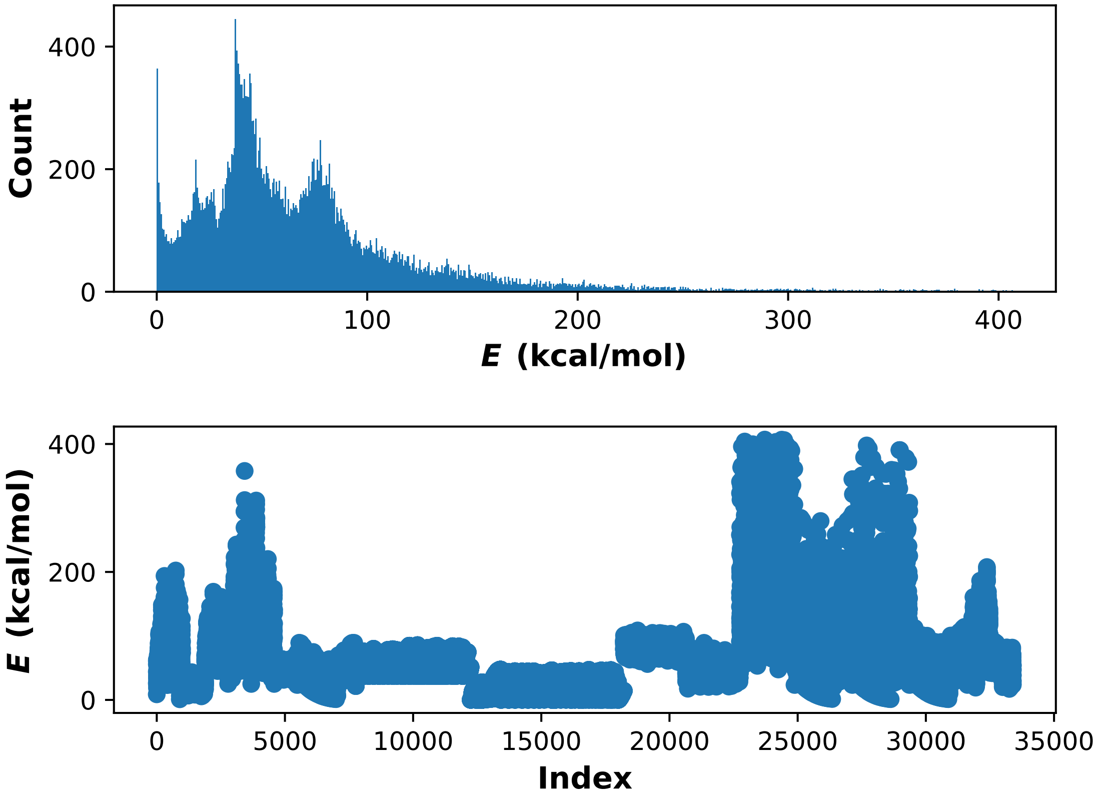

Figure S3: Energy distribution of the test data set.

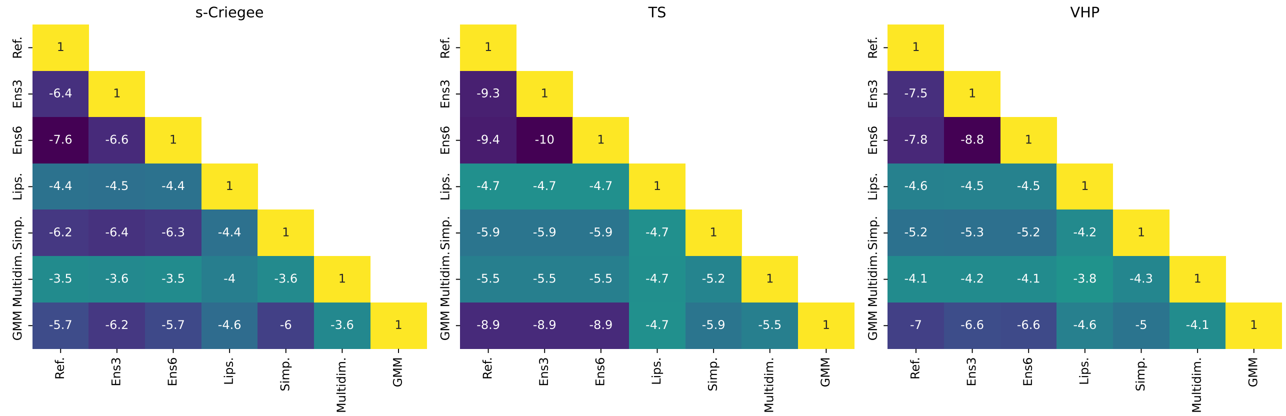

Figure S4: Root Mean Square Displacement of the stationary points (VHP, Transition State and S-Criegee) of the potential energy surface with respect to the *ab-initio* reference structure and between the different obtained geometries. Notice that the logarithm of the value of RMSD is reported to exemplify the differences between the values better.

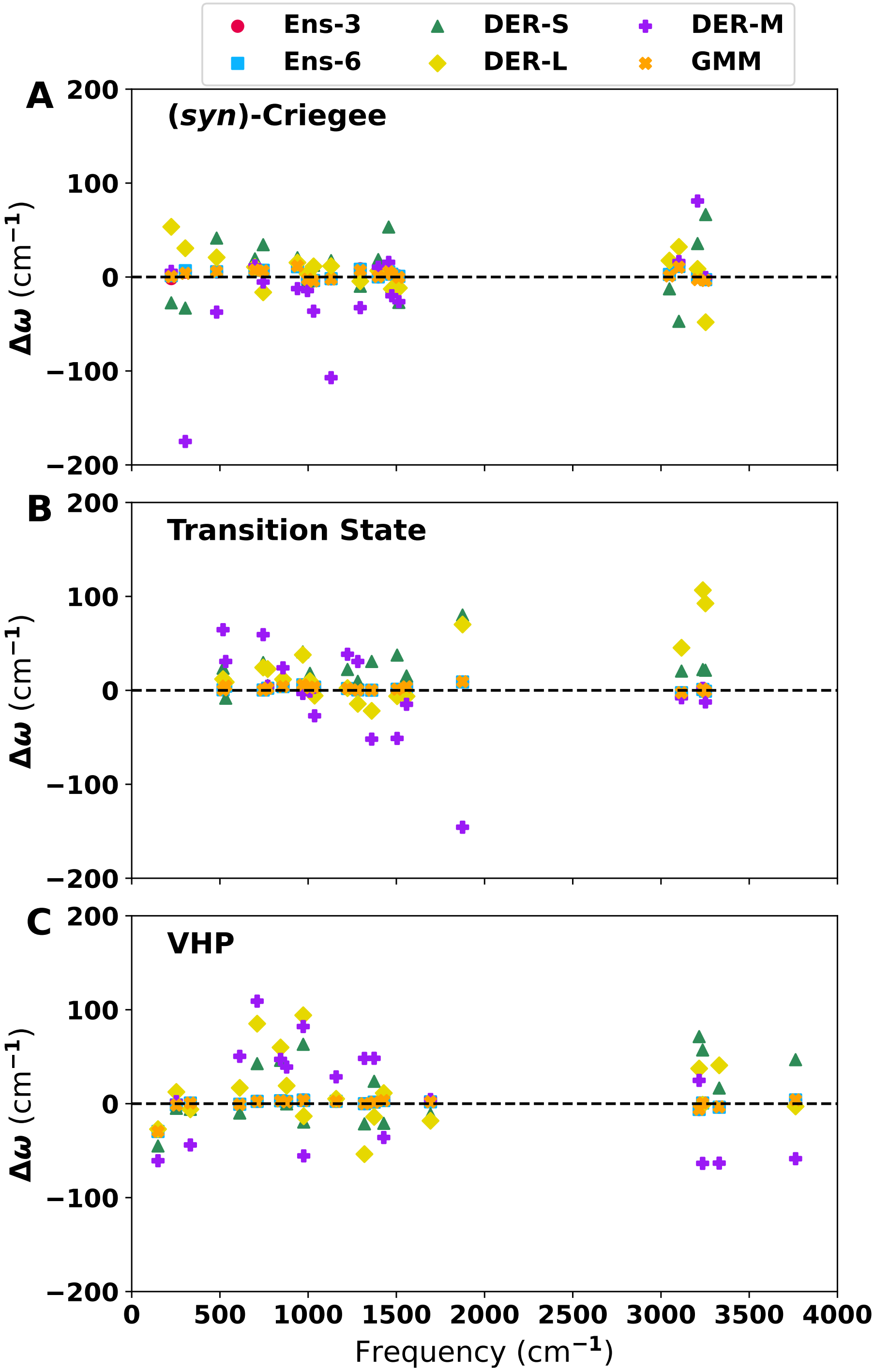

Figure S5: Error per predicted harmonic frequency ($\Delta\omega = \omega_{\mathrm{ref}} - \omega_{\mathrm{pred}}$) of the *(syn)*-Criegee (A), transition state (B) and VHP (C) for all the UQ methods evaluated in this work. The values of the frequencies are reported in Tables S2, S3, and S4.

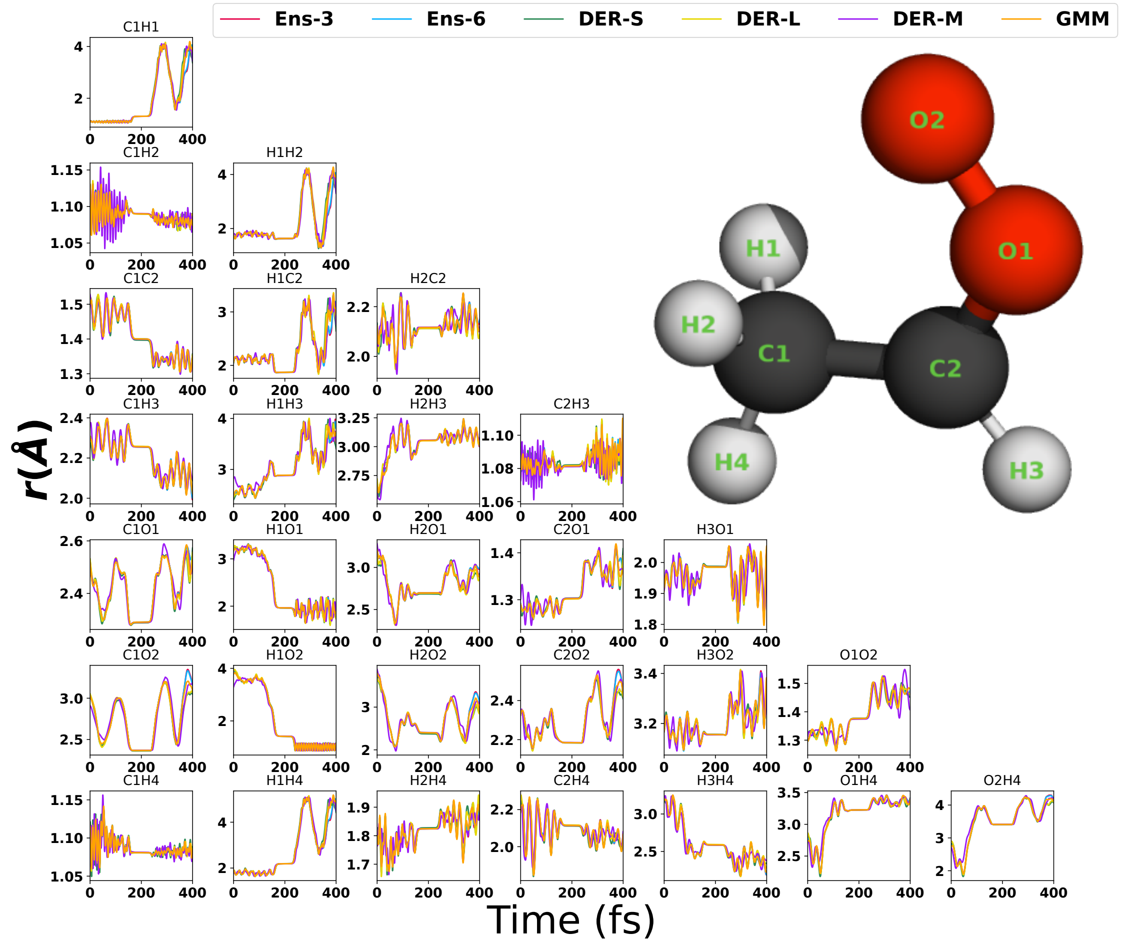

Figure S6: Atom-atom separation time series along the MDP for all models tested in this work. Each panel reports the distance between two atoms. The inset molecule displays the labelling of the atoms.

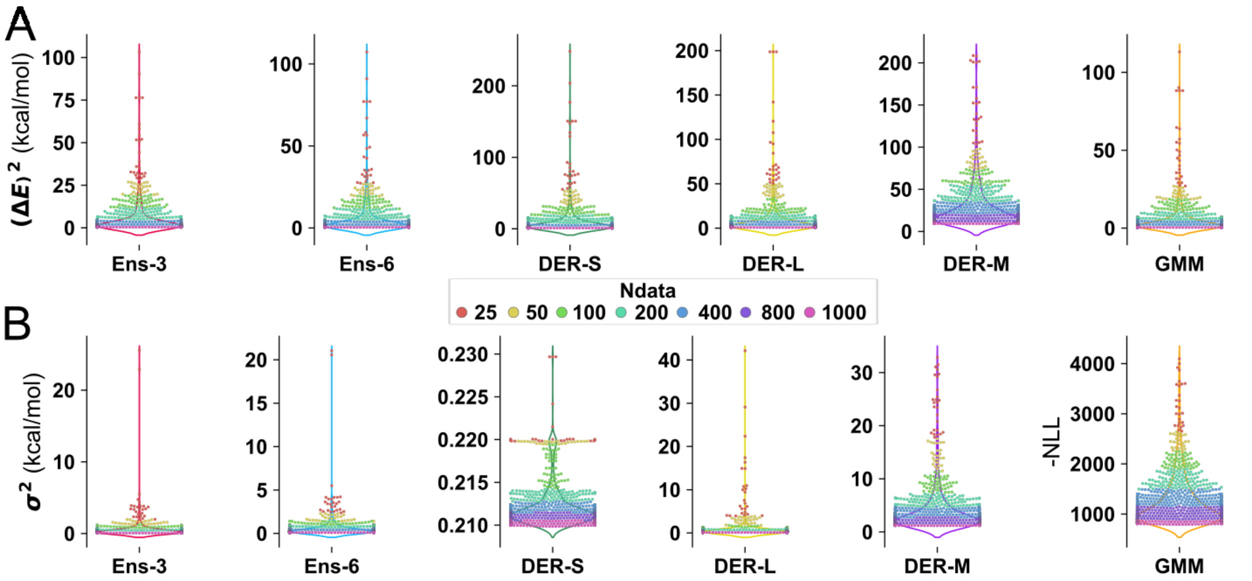

Figure S7: **Distribution of outliers as a function of variance and squared error.** Panels A and B show the violin plot of the 1000 samples with the highest variance (Panel A) or squared error (Panel B) from Figures 6 and 7. The points inside represent the location in the distribution of the different points and are coloured according to the range in which they can be found. Notice that for each model in panels A and B a different scale is used.

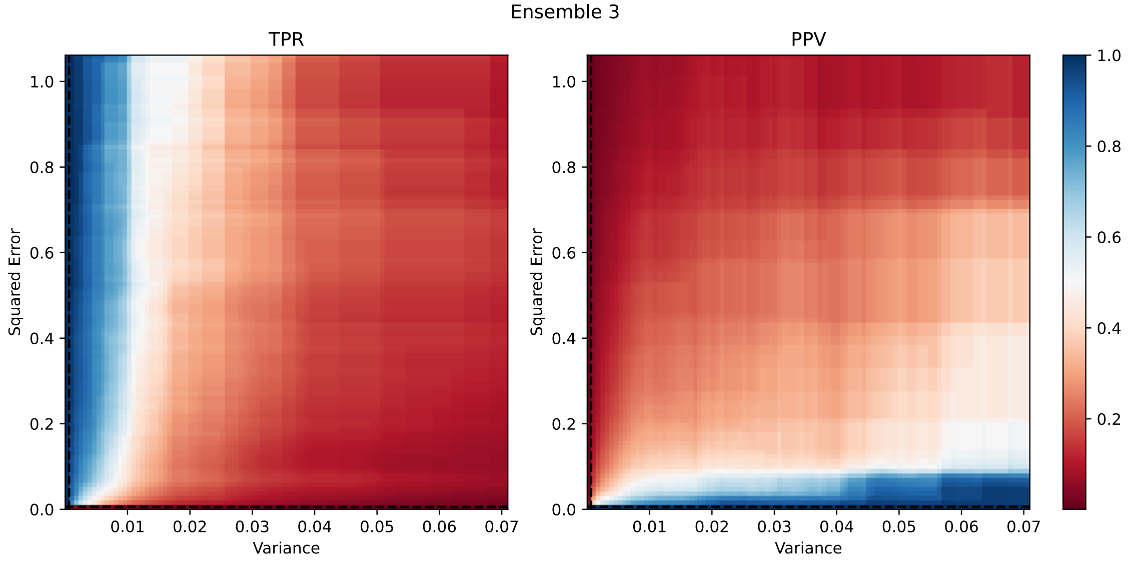

Figure S8: True Positive Rate (Left) and Positive Predictive Value (Right) for the Ens-3 model.

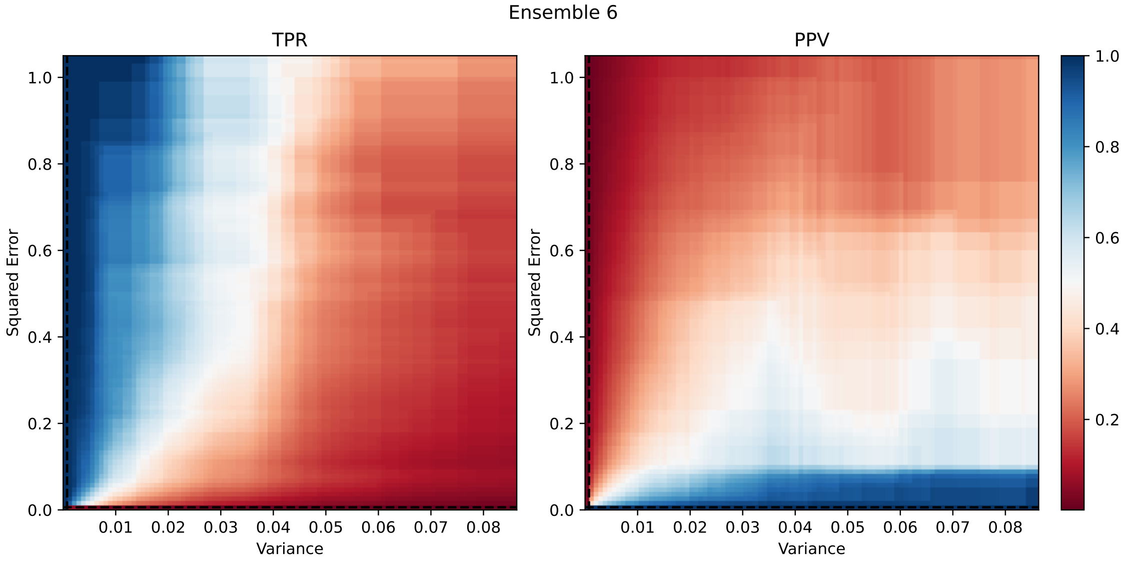

Figure S9: True Positive Rate (left) and Positive Predictive Value (right) for the Ens-6 model.

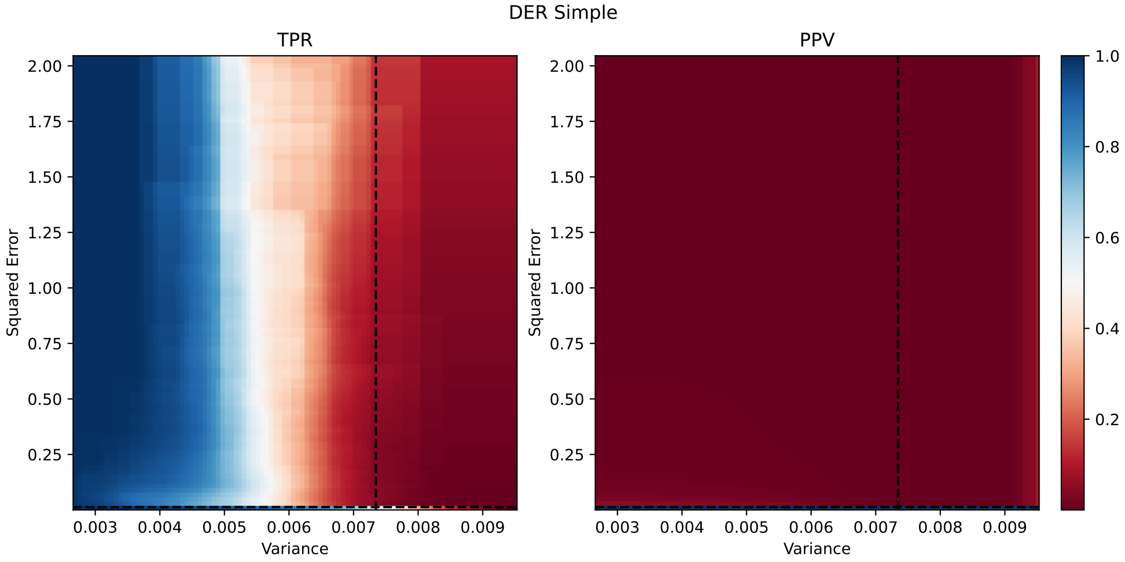

Figure S10: True Positive Rate (left) and Positive Predictive Value (right) for DER-S.

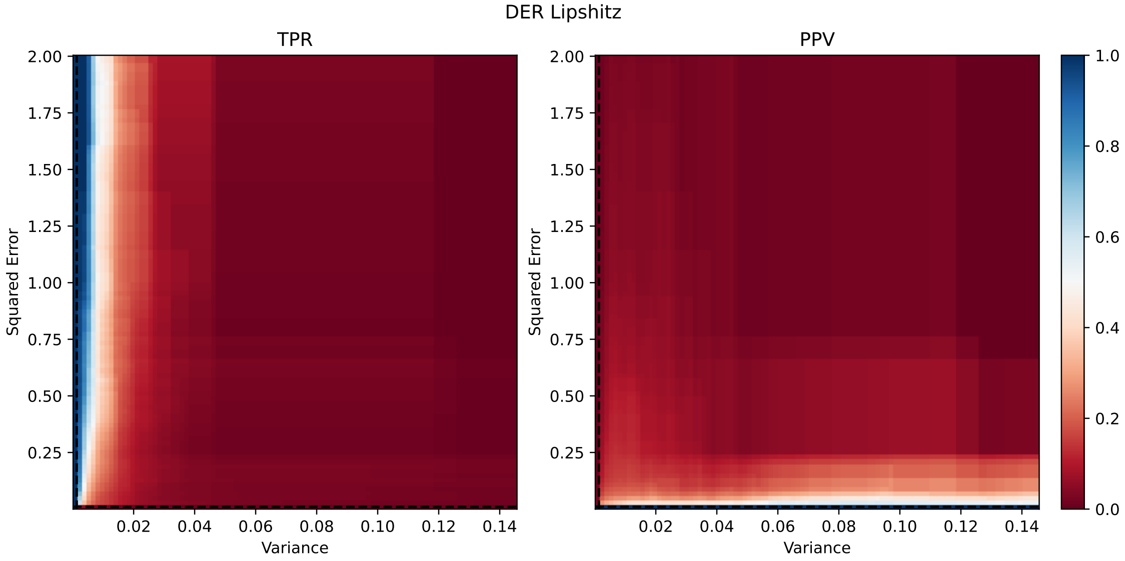

Figure S11: True Positive Rate (left) and Positive Predictive Value (right) for DER-L.

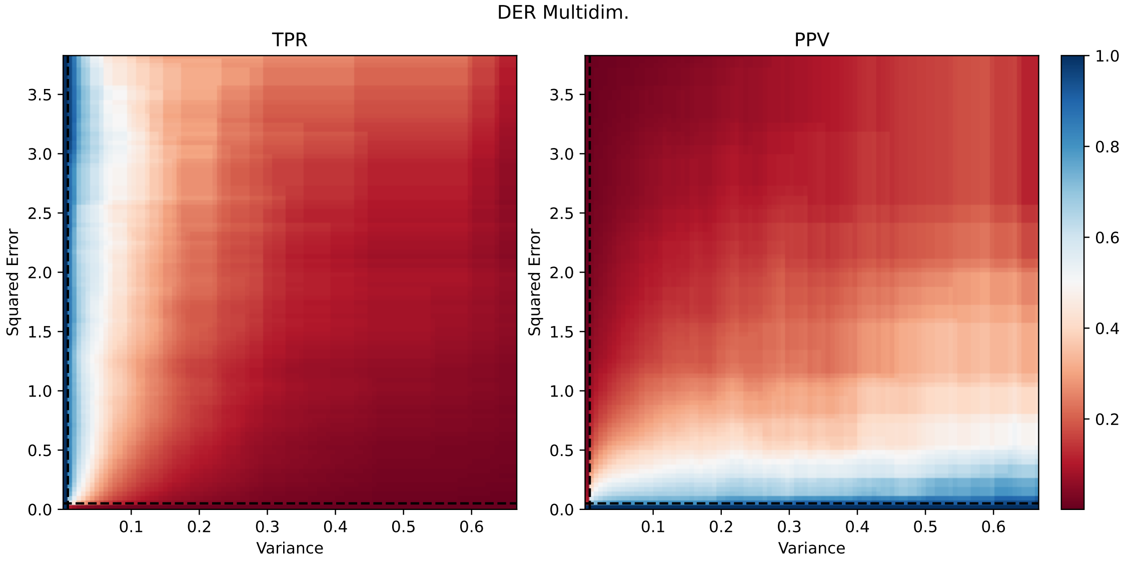

Figure S12: True Positive Rate (left) and Positive Predictive Value (right) for DER-M.

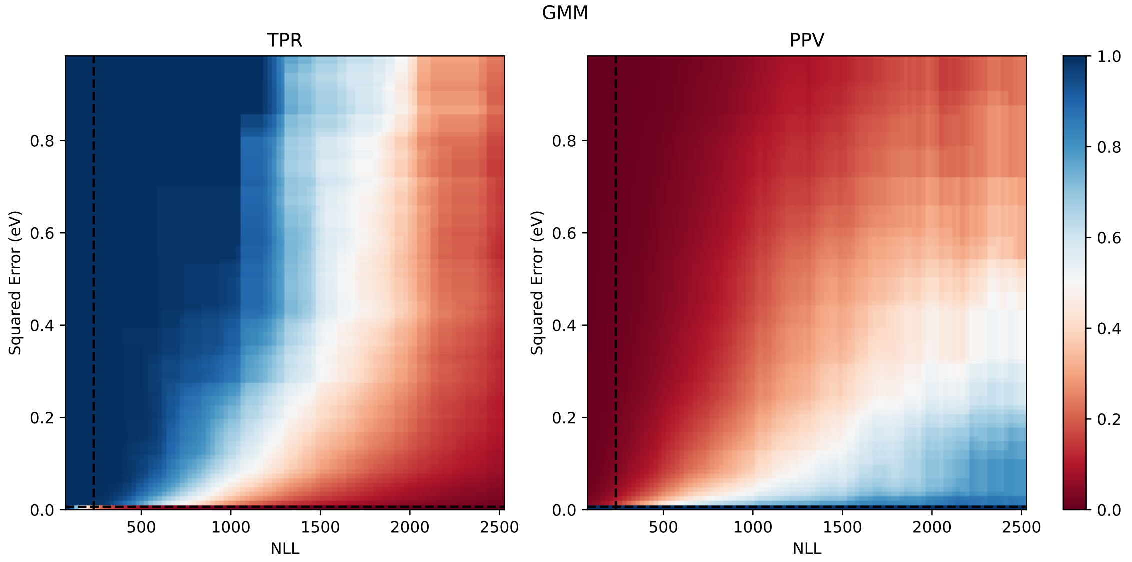

Figure S13: True Positive Rate (left) and Positive Predictive Value (right) for Gaussian Mixture Model.

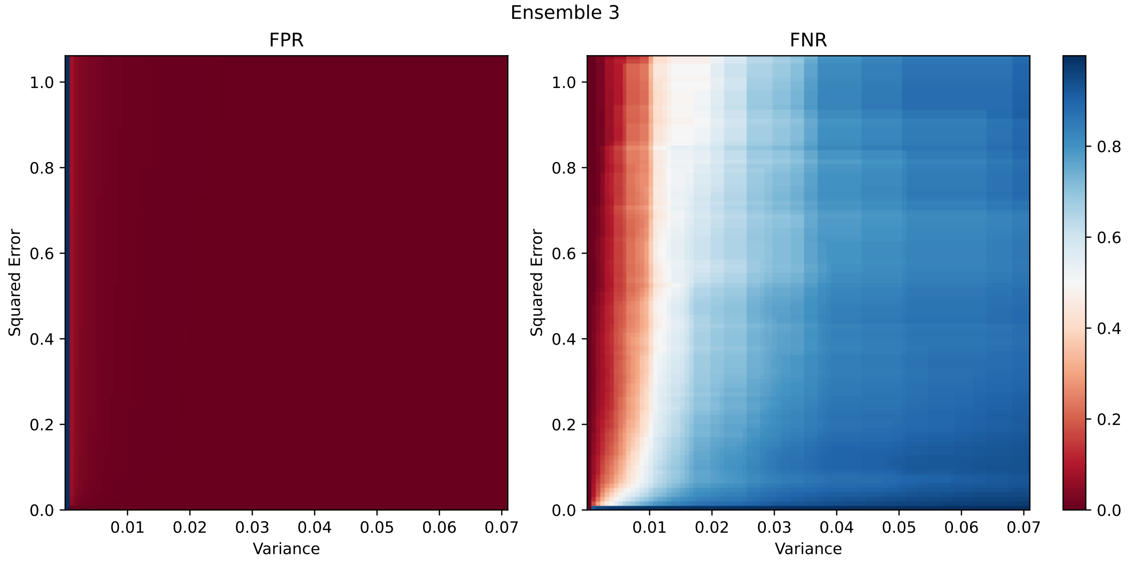

Figure S14: False Positive Rate (left) and False Negative Rate (right) for the Ens-3 model

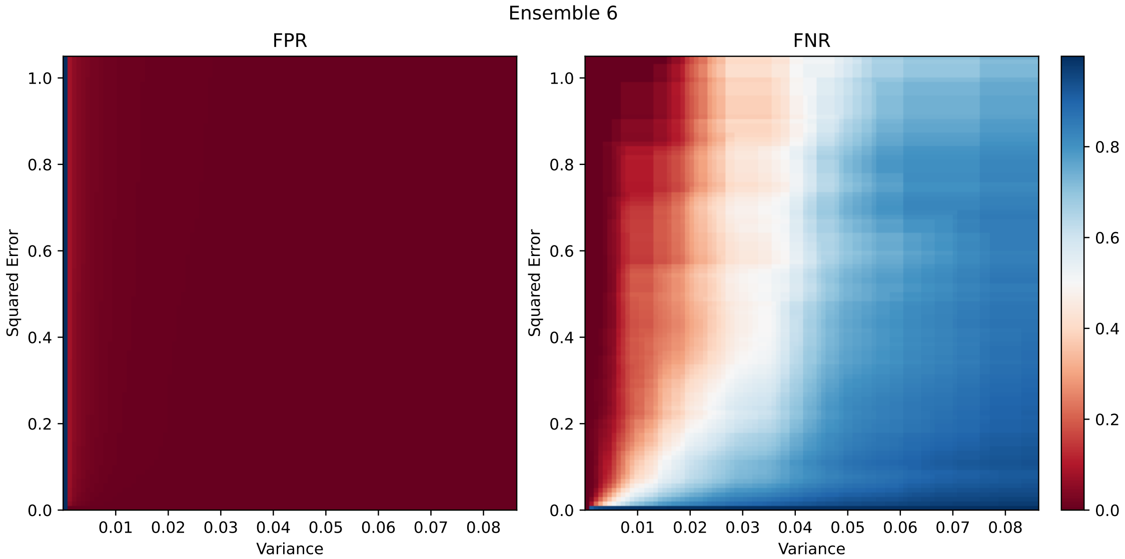

Figure S15: False Positive Rate (left) and False Negative Rate (right) for the Ens-6 model

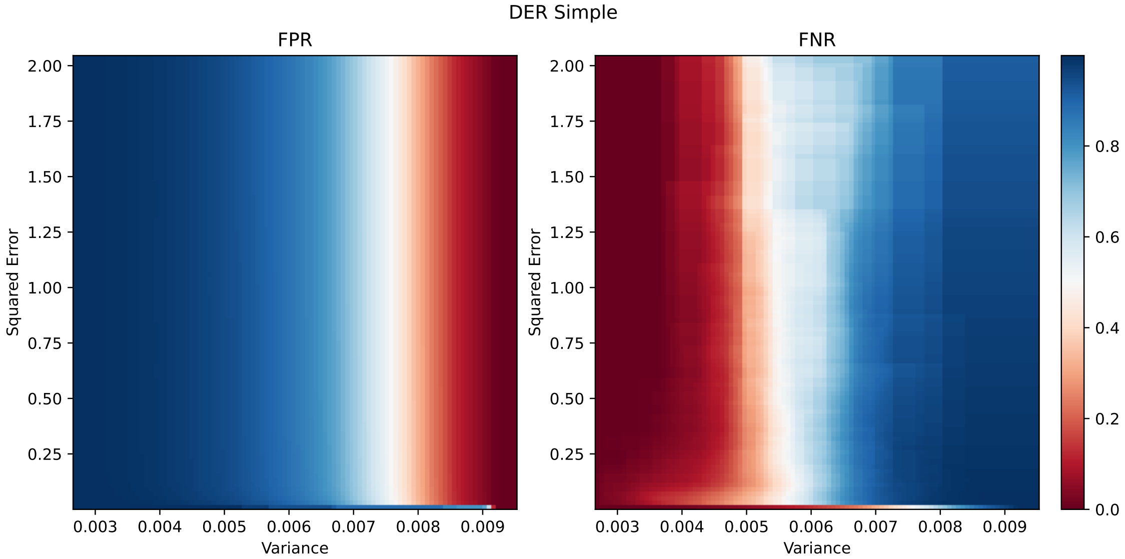

Figure S16: False Positive Rate (left) and False Negative Rate (right) for DER-S

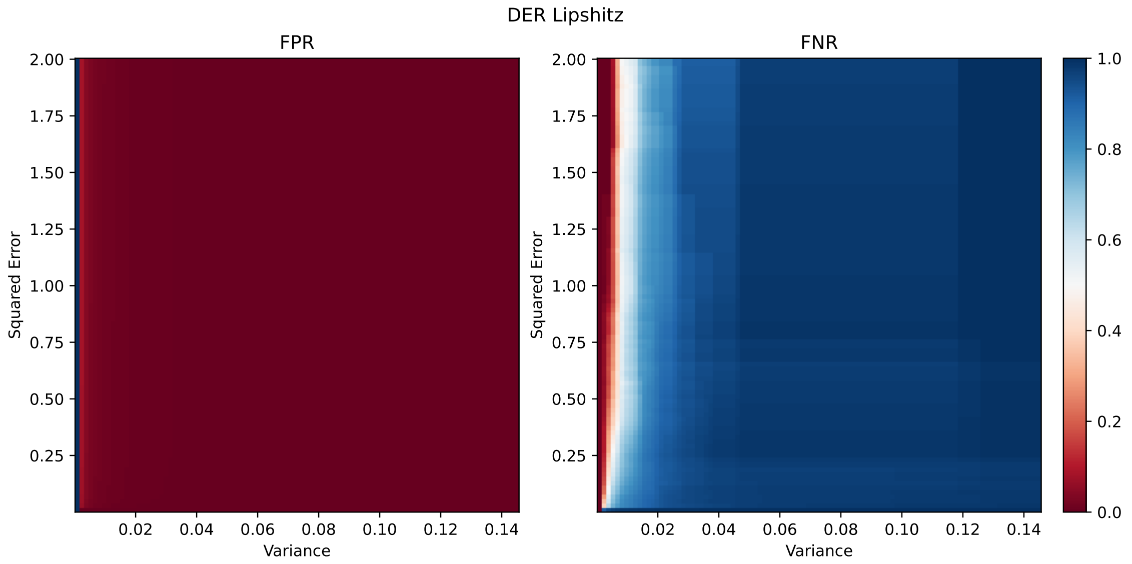

Figure S17: False Positive Rate (left) and False Negative Rate (right) for DER-L

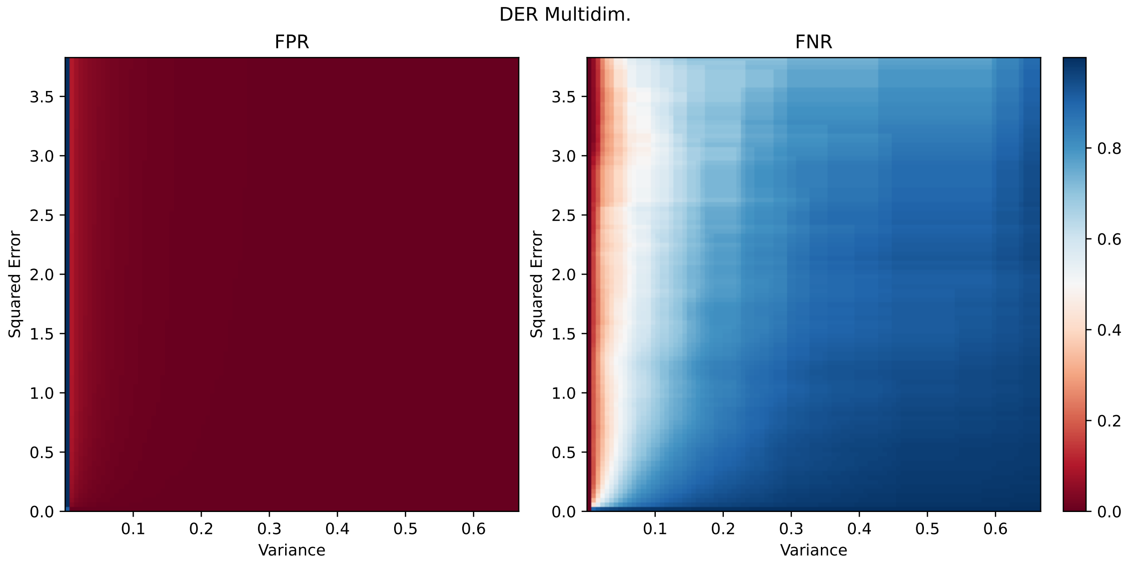

Figure S18: False Positive Rate (left) and False Negative Rate (right) for DER-M

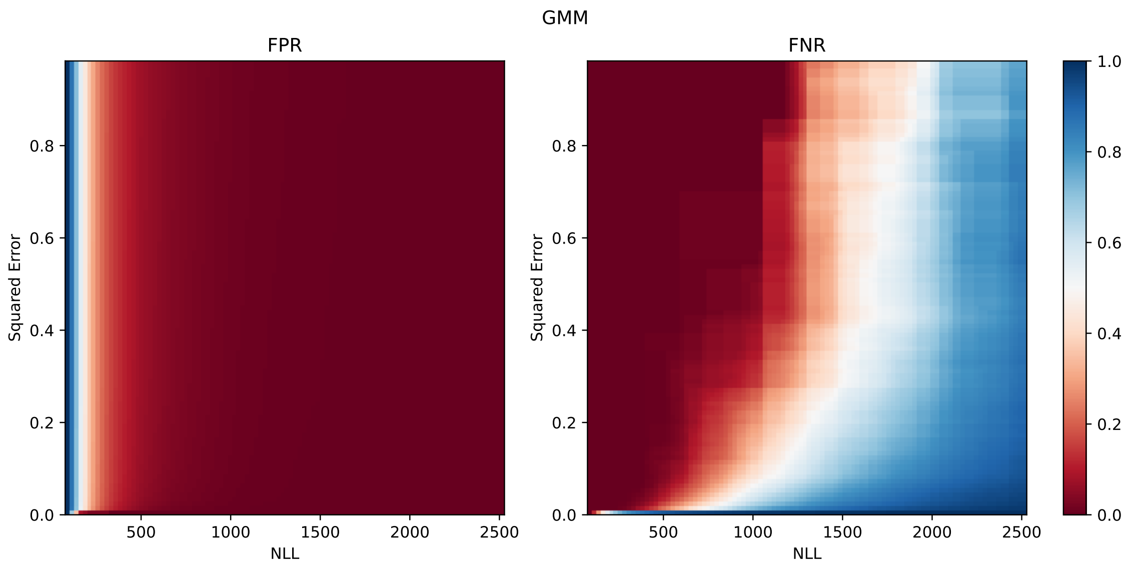

Figure S19: False Positive Rate (left) and False Negative Rate (right) for Gaussian Mixture Model

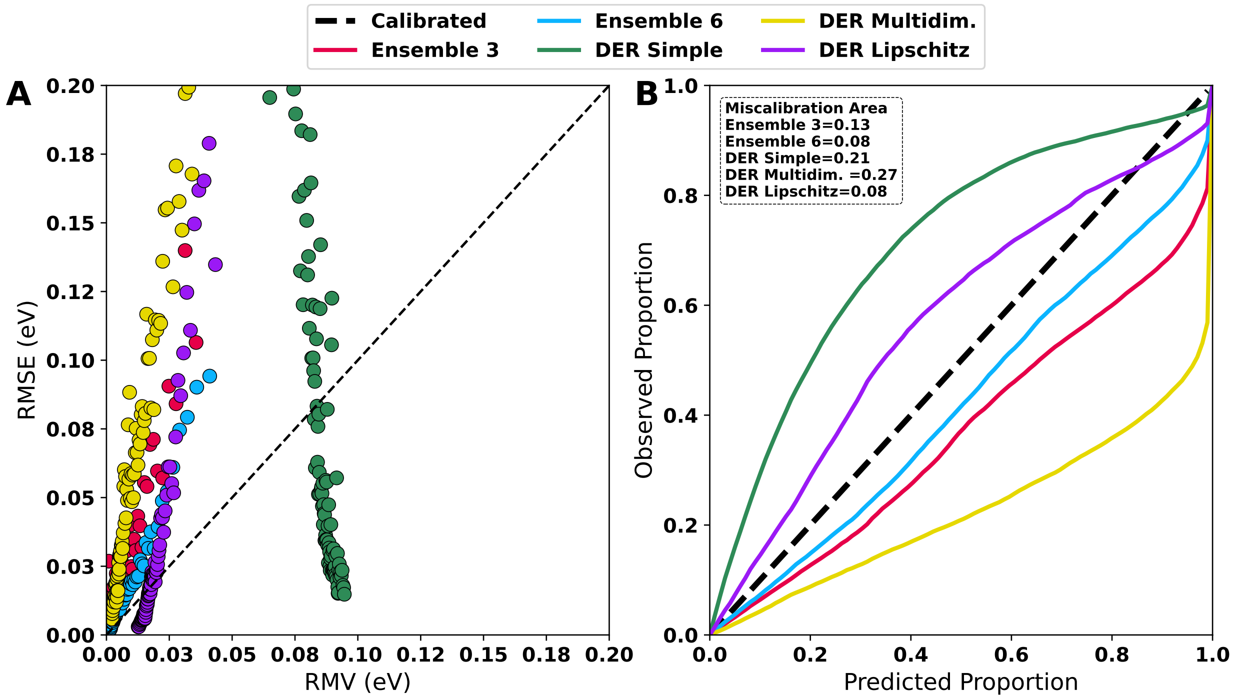

Figure S20: Metrics for calibration of the models. Panel A shows the empirical root mean squared error (RMSE) compared with the root mean variance (RMV) for the samples in the test set. Panel B shows the calibration curves of the models. The x-axis shows the predicted probability of obtaining the correct value for the error in a given percentile, and the y-axis shows the true probability. The insight box shows the miscalibration area (e.g. The area between the calibration curve and the diagonal representing perfect calibration)

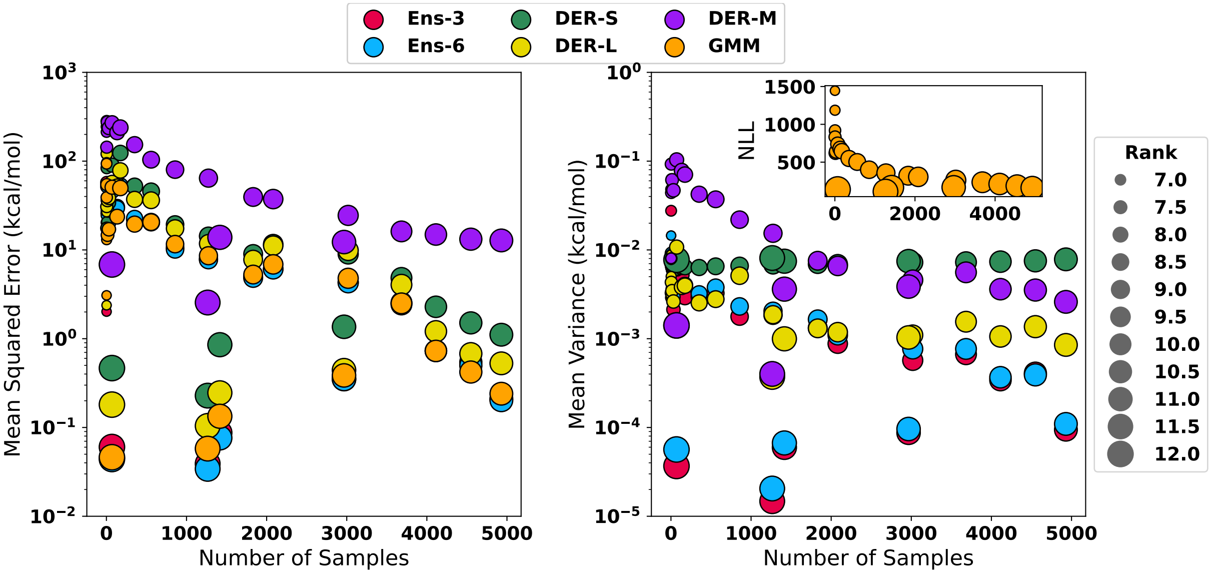

Figure S21: Changes in the mean square error (left) and mean variance (right) with respect to the number of samples in each class. The size of the scatter point is scaled with the ranking number. For the GMM model, the NLL is used to estimate the uncertainty. Notice that the y-axis scale is logarithmic.

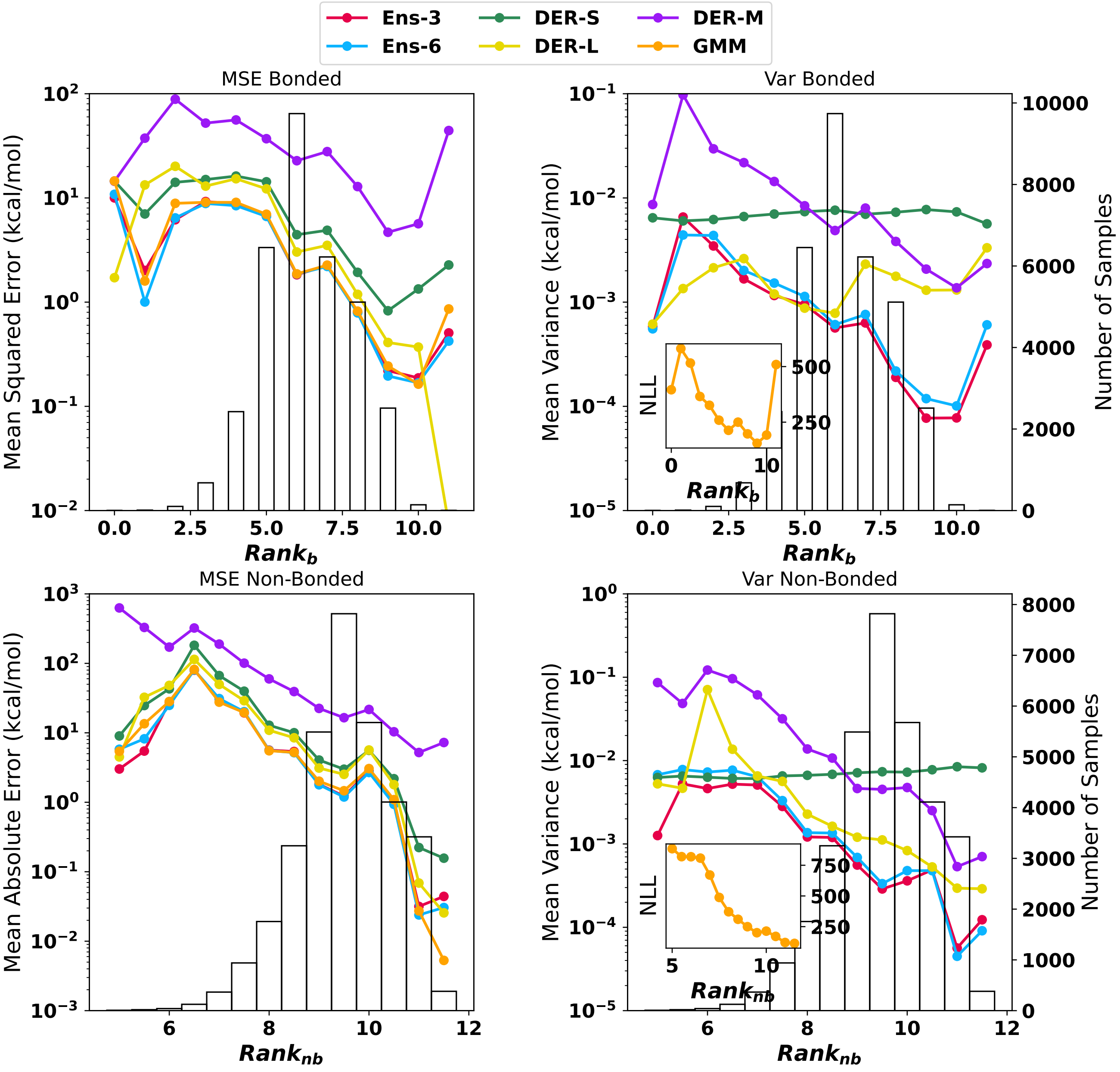

Figure S22: Changes in the mean square error (left) and mean variance (right) with respect to the rank of the molecules in the test set divided by contributions to bond (top) and non-bonded (bottom). In the background, a histogram of the number of samples with the same rank. For the GMM model, the NLL is used to estimate the uncertainty; therefore, the inset shows the changes in the NLL with respect to the rank. Notice that the y-axis scale is on logarithmic units.

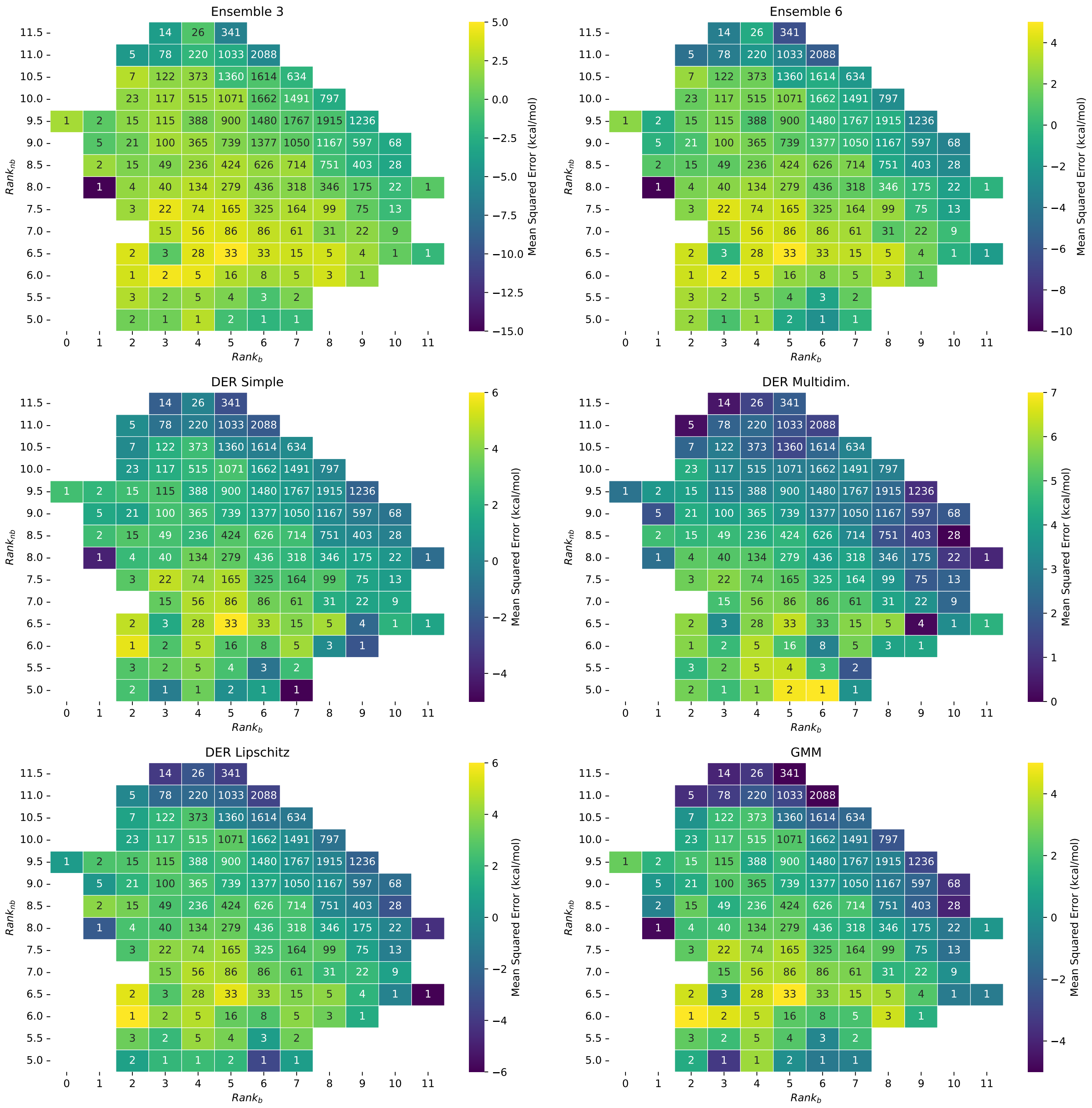

Figure S23: Map of influence of rank values for in and outside distribution of bond and non-bonded distances with respect to the error. The colour bar indicates the logarithm of the Mean Square Error and is normalised to its minimum and maximum values. The numbers inside each box are the number of samples for that score. The box is empty if no samples were found with that combination.

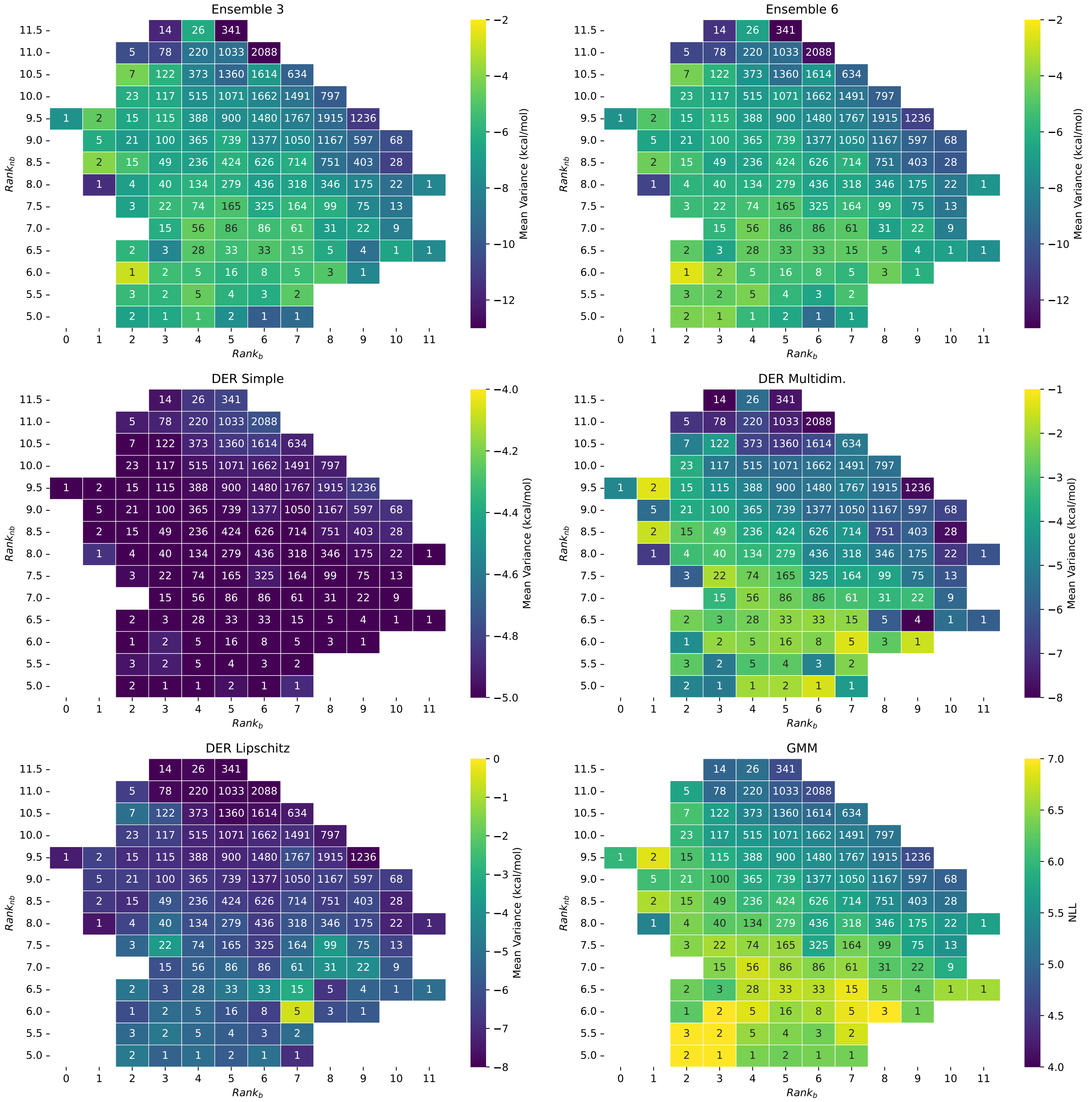

Figure S24: Map of influence of rank values for in and outside distribution of bond and non-bonded distances with respect to the variance. The colour bar indicates the logarithm of the mean variance except for GMM, which shows the NLL and is normalised to its minimum and maximum values. The numbers inside each box are the number of samples for that score. The box is empty if no samples were found with that combination.

References

(S1) Levi, D.; Gispan, L.; Giladi, N.; Fetaya, E. Evaluating and calibrating uncertainty prediction in regression tasks. *Sensors* **2022**, *22*, 5540.

(S2) Vazquez-Salazar, L. I.; Boittier, E. D.; Meuwly, M. Uncertainty quantification for predictions of atomistic neural networks. *Chem. Sci.* **2022**, *13*, 13068–13084.

(S3) Tran, K.; Neiswanger, W.; Yoon, J.; Zhang, Q.; Xing, E.; Ulissi, Z. W. Methods for comparing uncertainty quantifications for material property predictions. *Mach. Learn.: Sci. Technol.* **2020**, *1*, 025006.

(S4) Wang, J.; Manivasagam, S.; Wilson, A. K. Multireference character for 4d transition metal-containing molecules. *J. Chem. Theory Comput.* **2015**, *11*, 5865–5872.
